# Androgen receptor variant-7 regulation by tenascin-c induced src activation

**DOI:** 10.1186/s12964-022-00925-0

**Published:** 2022-08-10

**Authors:** Rintu Thomas, John Michael Jerome, Truong D. Dang, Eric P. Souto, Joshua N. Mallam, David R. Rowley

**Affiliations:** 1 Department of Molecular and Cellular Biology, Baylor College of Medicine, Houston, TX USA; 2 Lester and Sue Smith Breast Center, Baylor College of Medicine, Houston, TX USA

**Keywords:** Castration resistant prostate cancer, Preosteoblast, Tenascin-C, Androgen receptor variant 7 (AR-V7), Src activation

## Abstract

**Background:**

Bone metastatic prostate cancer does not completely respond to androgen-targeted therapy and generally evolves into lethal castration resistant prostate cancer (CRPC). Expression of AR-V7- a constitutively active, ligand independent splice variant of AR is one of the critical resistant mechanisms regulating metastatic CRPC. TNC is an extracellular matrix glycoprotein, crucial for prostate cancer progression, and associated with prostate cancer bone metastases. In this study, we investigated the mechanisms that regulate AR-V7 expression in prostate cancer cells interacting with osteogenic microenvironment including TNC.

**Methods:**

Prostate cancer/preosteoblast heterotypical organoids were evaluated via immunofluorescence imaging and gene expression analysis using RT-qPCR to assess cellular compartmentalization, TNC localization, and to investigate regulation of AR-V7 in prostate cancer cells by preosteoblasts and hormone or antiandrogen action. Prostate cancer cells cultured on TNC were assessed using RT-qPCR, Western blotting, cycloheximide chase assay, and immunofluorescence imaging to evaluate (1) regulation of AR-V7, and (2) signaling pathways activated by TNC. Identified signaling pathway induced by TNC was targeted using siRNA and a small molecular inhibitor to investigate the role of TNC-induced signaling activation in regulation of AR-V7. Both AR-V7- and TNC-induced signaling effectors were targeted using siRNA, and TNC expression assessed to evaluate potential feedback regulation.

**Results:**

Utilizing heterotypical organoids, we show that TNC is an integral component of prostate cancer interaction with preosteoblasts. Interaction with preosteoblasts upregulated both TNC and AR-V7 expression in prostate cancer cells which was suppressed by testosterone but elevated by antiandrogen enzalutamide. Interestingly, the results demonstrate that TNC-induced Src activation regulated AR-V7 expression, post-translational stability, and nuclear localization in prostate cancer cells. Treatment with TNC neutralizing antibody, Src knockdown, and inhibition of Src kinase activity repressed AR-V7 transcript and protein. Reciprocally, both activated Src and AR-V7 were observed to upregulate autocrine TNC gene expression in prostate cancer cells.

**Conclusion:**

Overall, the findings reveal that prostate cancer cell interactions with the cellular and ECM components in the osteogenic microenvironment plays critical role in regulating AR-V7 associated with metastatic CRPC.

**Video Abstract**

**Supplementary Information:**

The online version contains supplementary material available at 10.1186/s12964-022-00925-0.

## Background

Prostate cancer is the most common non-cutaneous cancer diagnosed in men. Androgen deprivation therapies (ADT) is the mainstay treatment for advanced prostate cancer. Although ADT produce significant initial clinical response, the efficacy is short-lived as majority of patients develop castration resistant prostate cancer (CRPC) [1, 2]. Cellular and functional heterogeneity in both the tumor and its microenvironment plays a critical role in both development and maintenance of CRPC. Thus, it is imperative to understand how tumor intrinsic and extrinsic factors interact and coordinate to facilitate therapeutic resistance so as to identify new targets to overcome CRPC [3, 4].

The stroma is critical for normal organ development and maintaining tissue homeostasis. The stroma of solid tumors is composed of both cellular components and non-cellular extracellular matrix (ECM) [5–11]. Tumor cells alter stromal cell type and the biochemical and mechanical properties of the ECM resulting in a reactive stroma phenotype. The reactive stroma is similar in organization and biology to a wound repair stroma and affects tumor growth and survival [7–9]. In prostate cancer, reactive stroma is a prognostic indicator for biochemical free recurrence following prostatectomy and for prostate cancer-specific death [8–13].

Tenascin-C (TNC), an ECM glycoprotein, is a critical component of the reactive stroma that is expressed during the earliest phase of prostate cancer development [6–8]. Apart from cancer, TNC is expressed de novo in adults at sites of active tissue repair, during infections, and chronic inflammations [15]. TNC promotes tumor growth by directly influencing intracellular signaling activating tumor proliferation, survival, and epithelial to mesenchymal transition (EMT) [16]. Indirectly, TNC affects tumor growth by influencing the stem cell niche in the tumor microenvironment and by inducing angiogenesis and suppressing the immune system [6, 14–18]. TNC expression is associated with metastatic fitness and poor clinical outcome in several malignancies including prostate, breast, lung, colorectal, head and neck, and melanoma [13, 16, 19–23]. These findings suggest the vital role of TNC in both tumor initiation and tumor progression.

TNC is also expressed in metastatic niches such as the bone, lungs, lymph nodes, and functions as a chemoattractant providing a suitable milieu for disseminated tumor cells to survive and form overt tumors [22, 24, 25]. Analysis of prostate cancer bone metastases samples indicate tumor formation in the osteogenic niche of the bone endosteum that is rich in TNC [24]. Bone metastatic prostate cancer cells were observed to differentially adhere and proliferate on TNC in both in vitro organoid cultures and in vivo models [24]. Together, these data suggest that TNC in the reactive endosteum may be important for establishing metastatic tumor foci and for bone metastatic prostate cancer progression. Mechanisms, however, remain unknown.

Approximately 90% of prostate cancer patients die of bone metastases [26]. Androgen Receptor (AR) is a critical driver of prostate cancer metastases and sustains metastatic disease; however, the majority of bone metastases are castration resistant and unresponsive to AR targeted therapy [27–29]. The emergence of constitutively active AR splice variant 7 (AR-V7) with a truncated ligand binding domain is a critical resistant mechanism associated with metastatic CRPC. AR-V7 expression is negligible in primary prostate tumor, but expression increases in patients receiving ADT [30]. Importantly, both high transcript and protein levels of AR-V7 are detected in prostate bone metastases and are associated with shorter patient survival [31, 32]. Although TNC affects bone metastatic prostate cancer, little is known about potential interactions between TNC-induced biology and the evolution of AR-V7-regulated biology. Accordingly, elucidating the mechanism of AR-V7 regulation in the context of the bone microenvironment, including TNC, may aid in developing strategies to avert therapeutic resistance. Moreover, understanding how hormone agonists and antagonists affect this biology will help elucidate mechanisms.

In the present study, we developed heterotypical organoid models of human prostate cancer- osteoblast interactions to more precisely dissect gene expression and mechanisms of action. We report here that both *TNC* and *AR-V7* expression was induced in prostate cancer cells due to interaction with preosteoblasts. This expression was repressed by testosterone and was upregulated by estradiol and enzalutamide. Importantly, TNC functioned to stabilize AR-V7 protein post-translationally via Src activation. In a reciprocal manner, AR-V7 functioned to upregulate *TNC* expression. Furthermore, inhibition of Src kinase activity resulted in downregulation of both *AR-V7* splicing and *TNC* expression in prostate cancer cells. In this context, understanding the link between bone microenvironment niche proteins such as TNC, and Src dependent AR-V7 activity provides mechanistic insight as to how this microenvironment may contribute to the evolution of CRPC. These findings may also provide insight towards therapeutic targets for AR-V7 positive, bone metastatic prostate cancer.

## Methods

### Cell culture

Human prostate cancer cells VCaP and murine preosteoblast cell line MC3T3-E1 were purchased from ATCC (Manassas, VA). In addition, 22Rv1 was also used for experiments. VCaP was cultured in high glucose DMEM media (Gibco, #11965–084); 22Rv1 was cultured in RPMI 1640 media (Gibco, #11875–093); MC3T3-E1 sub clone 4 was cultured in MEMα media (Gibco, #A10490-01). The basal media for all cell lines was supplemented with 10% fetal bovine serum and 1% Penicillin/Streptomycin antibiotic cocktail. LNCaP^AR−V7/pLenti^ was cultured in RPMI 1640 media supplemented with 10% FBS and Geneticin-G418 (ThermoFisher Scientific, Waltham, MA # 10131035) at a working concentration of 350 μg/ml. Addition of Doxycycline (Sigma Aldrich, St. Louis, MO # D3447) induced AR-V7 expression. All cell lines were authenticated using short tandem repeat profiling through LabCorp (Burlington, NC) and tested regularly for mycoplasma.

### Generation of stable prostate cancer cell lines

VCaP and 22Rv1 were transduced with *pLL-CMV-rFLuc-T2A-GFP-mPGK-Puro* from System Biosciences, Palo Alto, CA (#LL310VA-1) to generate stable cells expressing GFP and red firefly luciferase (rFLuc) via puromycin selection (ThermoFisher Scientific, # A1113803) at 3ug/ml for VCaP and 5ug/ml for 22Rv1. MC3T3-E1 was transduced with *pLL-CMV-RFP-T2A-Puro* (System Biosciences,#LL110VA-1) to generate stable cells expressing RFP selected using puromycin at 3ug/ml.

### Culturing cancer cells on TNC

Both 12 and 6 well tissue culture plates were coated with purified human TNC (Sigma # CC065) at a concentration of 75 μg/ml/0.19 cm^2^ based on published protocols [24]. Bovine serum albumin ((BSA)-Sigma# A8806) dissolved in PBS was used as a control. The TNC and BSA coated plates were incubated in 37 °C overnight until the coating was dry.

VCaP and 22Rv1 seeded on BSA or TNC coated plates were maintained in 5% charcoal-stripped serum (csFBS) base media. Cells were seeded at a density of 2.0E5 cells/well in 12 well plates and 4.0E5/well in 6 well plates for 72 h. RNA was extracted for analyzing gene expression and protein extracted for conducting Western blots. Cells used for confocal imaging were plated onto μ-Slide 2 Well ibiTreat slides (Ibidi, WI #80286) coated with BSA or TNC as mentioned above.

### Compounds

Anti-Tenascin antibody was used (BC-24, Sigma#SAB4200782) and AR-V7 protein level was assessed in VCaP and 22Rv1 using Western blot and confocal microscopy. The antibody isotype (#5415) from Cell Signaling Technology (CST), MA was used as a control. TNC-induced post-translational stability was assessed using cycloheximide chase assay based on published protocol [33]. Src inhibitor-PP1 was purchased from Sigma (#CAS 172889–26-8).

### RNA Isolation and RT-qPCR

Co-cultures of 22Rv1/VCaP and MC3T3-E1 were seeded at a density of 2.0E5 each to form heterotypical organoids in ultra-low attachment plates. Organoid cultures were maintained in MEMα media in 5% csFBS, and 1 × P/S. Organoids were treated with vehicle control (ethanol or DMSO), testosterone (1 nM), estradiol (10 nM), or enzalutamide (10 µM) for 72 h. RNA was collected using RNeasy Mini Kit following manufacturer’s protocol (Qiagen, #74106). Same protocol for RNA extraction was also applied for 2D and 3D cancer cultures. Reverse transcription using amfiRivert cDNA Synthesis Platinum Master Mix (GenDEPOT, Katy, TX #R5600) followed by qPCR via ViiA 7 system (Applied Biosystems/Life Technologies) by using FastStart Universal SYBR Green reaction mastermix (Roche, #04913914001) was conducted. Primer specificity (human vs mouse) was validated using SuperScript One-Step RT-PCR System (Invitrogen #10928–042). Relative transcript levels were normalized to *RPL30* because variance in *RPL30* expression was negligible between experimental conditions. RT-qPCR was conducted for n = 3 biological replicates. Validated human specific primer sequences are listed in Additional file 1: Table S1.

### Western blot

Protein lysates were diluted in water with Laemmli buffer to a concentration of 1.5 μg/μl and separated on 6%, 8%, and 10% SDS-PAGE gels. The proteins were then transferred onto PVDF membrane followed by blocking in 5% milk dissolved in TBST. Blots were incubated in primary antibodies overnight at indicated dilutions followed by 1-h incubation in secondary antibody the next day. Antibodies used with dilutions are listed in Additional file 2: Table S2. PVDF blot was exposed to ECL Plus chemiluminescence reaction. Western blot densitometry analysis were conducted for n = 3 biological replicates using Image J and normalized to β-actin.

### siRNA knockdown assay

Cell lines were transfected with small interfering RNA (siRNA) oligonucleotides using lipofectamine RNAimax (ThermoFisher Scientific # 13778075). The siRNA sequences for AR-FL and AR-V7 were designed based on previous publication and designed through Dharmacon, Horizon Discovery, Cambridge, United Kingdom [32]. Silencer pre-designed siRNA for Integrin β1 (#109877) was purchased from ThermoFisher Scientific in addition to control/scrambled siRNA. The siRNA target sequence for Src is CGUCCAUAUUUAACAUGUAUU and designed through Dharmacon, Horizon Discovery.

### Immunocytochemistry (ICC)

Cells were fixed with 4% PFA and permeabilized using 0.5% Triton X-100 in PBS for 15 min, and blocked in 1% goat serum in PBS for 1 h. The cells were incubated overnight at 4^0^C with AR-V7 antibody. The cells were washed and incubated with secondary antibody for 1 h and nuclei were counterstained with DAPI. VCaP and 22Rv1 cells plated on TNC were treated with anti-tenascin antibody or isotype control then fixed and stained for AR-V7 using the same protocol. The specificity of AR-V7 antibody used in ICC was validated in LNCaP^AR−V7/pLenti^ engineered with doxycycline inducible AR-V7 (Additional file 5: Fig. S3C) [34]. Images were acquired through Nikon A1-R confocal microscope. The exported images were analyzed using Image J. The fraction of AR-V7 positive nuclei in BSA versus TNC coated slides were quantified by converting the fluorescent images to binary images followed by setting up the threshold. AR-V7 positive nuclei within the set threshold are counted against the total number of nuclei (DAPI stained) in the field of interest. The method was repeated for n = 3 independent replicates and student t-test conducted to determine statistical significance. Integrated fluorescence intensity of AR-V7 was measured using Image J. The integrated fluorescence intensity measurement was repeated for n = 3 biological replicates and student t-test conducted to determine statistical significance.

### 3D Organoid fixing, staining, and confocal imaging

Co-cultures of MC3T3-E1-RFP with 22Rv1-GFP/Luc or VCaP-GFP/Luc were seeded at a density of 2.0E5 total cells/cm^2^ to form heterotypical organoids on millicell-CM cell culture inserts (#PICM01250) from Sigma. TNC antibody was conjugated to FITC (abcam # ab188285) based on recommended protocol. Organoid culture, fixing and staining protocol were adapted and modified from published protocols [35, 36] and images were acquired through Nikon-A1 confocal microscope. Z-stacks of the images were then utilized for 3D rendering of the images using Imaris software.

#### Statistical analysis

Statistical analyses were performed using GraphPad Prism 7 software. Unpaired and two-tailed students t-tests were performed where appropriate. Statistical significance was accepted at the 95% confidence level (*p<0.05, ** p<0.01, ***p<0.001, ****p<0.0001).

## Results

### TNC expression and deposition in heterotypical organoids.

**Fig. 1:**
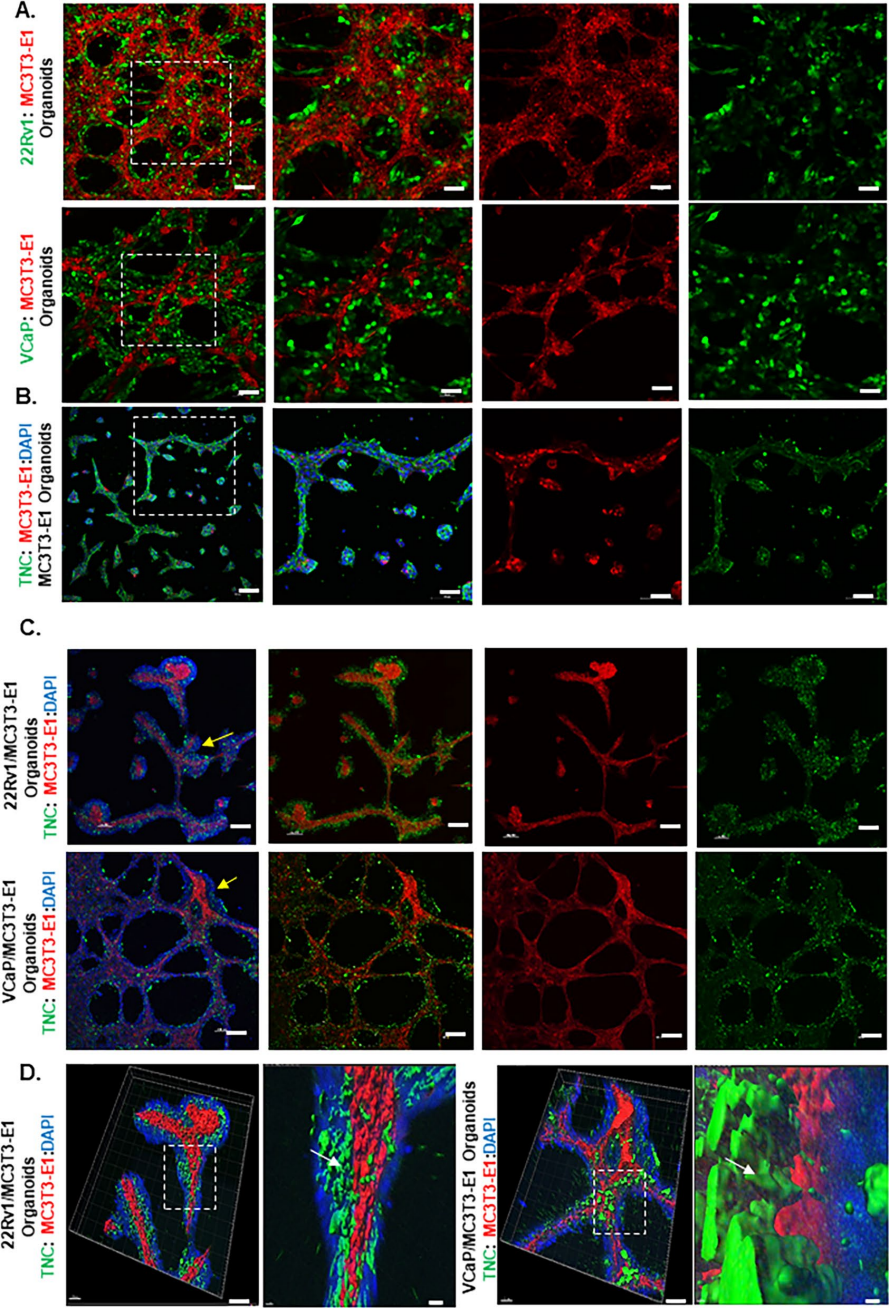
TNC deposition in regions of contact between mouse preosteoblasts and prostate cancer cells in 3D heterotypical organoids. **A** Compartmentalization of preosteoblasts (RFP) and prostate cancer cells (VCaP and 22Rv1-GFP) in 3D heterotypical organoids (Scale bars, 10x: 100 µm and 20x:50 µm). **B** TNC (FITC) expression by preosteoblasts in 3D organoids (Scale bars, 10x:100 µm and 20x:50 µm). Nuclei are counterstained with DAPI. **C** TNC staining (FITC) on 3D heterotypical organoids composed of VCaP and 22Rv1 (yellow arrow: depicted with DAPI) and MC3T3-E1 (RFP) (Scale bar, 10x: 100 µm). **D** 3D reconstruction using Imaris of 20 × confocal images of 3D heterotypical organoids depicting TNC deposition (green-white arrow) on regions of contact between prostate cancer cells (DAPI) and MC3T3-E1 (RFP) (Scale bars, 20x:50 µm, Imaris: 20 µm)

Heterotypical organoids were developed and composed of GFP-labeled VCaP and 22Rv1 and RFP-labeled preosteoblasts. Organoids self-organized with an inner core of preosteoblasts and an outer layer of prostate cancer cells that colonize on the preosteoblasts (Fig. 1A). TNC was localized on the surface of preosteoblasts in organoids (Fig. 1B). In vivo, TNC expression is limited to the endosteal surface-a site of active bone repair [37, 38]. Our studies and others have shown that prostate cancer metastasizes to a TNC rich osteogenic niche of the bone [24, 27]. Interestingly, 3D rendering of heterotypical organoids showed TNC in regions of contact between prostate cancer cells and preosteoblasts (Fig. 1C, D).

### Hormone regulation of bone repair-associated genes

Both testosterone and estradiol regulate bone homeostasis in males and females [39, 40]. We show that testosterone (1 nM) and estradiol (10 nM) differentially regulate expression of several genes involved in osteogenesis in preosteoblasts (Additional file 3: Fig. S1A). These include several bone development and repair-centric genes.

Similarly, interactions with preosteoblasts induced genes involved in osteo-mimicry/bone repair processes in VCaP and 22Rv1 (Table 1). Moreover, these genes were differentially regulated by testosterone, estradiol, and enzalutamide. This included bone morphogenetic proteins (*BMP*s), hedgehog ligands (Indian hedgehog-*IHH*, Sonic hedgehog-*SHH*) with its associated receptor patched 1 (*PTCH1*), and transforming growth factor-beta (*TGFβ*) (Table 1). Interestingly, the expression of these genes and others including downstream regulators of hedgehog signaling such as *Gli*s (*Gli1,Gli2, and Gli3*) were further stimulated by estradiol and enzalutamide and repressed by testosterone. Importantly, we excluded the gene expression from mouse-derived preosteoblasts in the organoid cultures using human specific primers (Additional file 1: Table S1). Analyses of both in vivo prostate-bone metastasis models and established human prostate bone metastases samples reveal a similar osteo-mimicry like gene signature in prostate cancer cells [41]. These data suggest that induction of genes involved in osteo-mimicry in prostate cancer cells may be permissive or promoting cancer cell survival in the bone microenvironment by inducing a repair phenotype. Further, analyses of human prostate bone metastasis microarrays reveal the bone metastatic niche to be high in TNC and pro-collagen1, which reflect active bone repair or regeneration [24, 42, 43].

**Table 1:** Differential human gene expression in 3D prostate cancer/preosteoblast heterotypical organoids treated with hormone agonists and anti-AR treatment

22Rv1 Upregulated										22Rv1 Downregulated							
MousePre-osteoblast		Testosterone1nM		Estradiol10nM			Enzalutamide10µM		MousePre-osteoblast			Testosterone1nM		Estradiol10nM		Enzalutamide10µM	
Genes	P-value	Genes	P-value	Genes	P-value	Genes		P-value	Genes		P-value	Genes	P-value	Genes	P-value	Genes	P-value
*BMP2*	9.86E-05	*ELF3*	1.98E-04	*BMP2*	1.11E-03	*BMP2*		1.50E-03	*ICAM1*		5.50E-03	*BMP2*	6.91E-06	*ICAM1*	2.27E-05	*BMP6*	4.02E-03
*IHH*	4.90E-02	*TGFβ1*	2.96E-02	*IHH*	1.90E-02	*IHH*		5.38E-03	*FGF9*		2.77E-02	*BMP6*	1.30E-02	*FGF9*	2.00E-02	*GlI1*	4.44E-04
*SOX9*	3.90E-02	*SOX9*	6.36E-04	*SHH*	1.14E-02	*ELF3*		2.96E-05				*IHH*	4.50E-02	*IL1α*	5.62E-04	*GlI2*	3.34E-04
*PTCH1*	2.17E-03	*ITGβ1*	2.00E-02	*SOX9*	7.13E-04	*PTCH1*		9.14E-04				*PTCH1*	7.61E-03	*CSF3*	7.60E-03	*ICAM1*	9.74E-03
*TGFβ1*	4.68E-04			*GlI1*	1.30E-03	*Gli3*		1.64E-02				*GlI1*	2.88E-04	*ESR1*	9.85E-04	*FGF9*	4.70E-04
*ELF3*	2.64E-03			*GlI3*	7.50E-03	*TGFβ1*		3.55E-03				*GlI2*	1.74E-02	*ESR2*	4.48E-03	*IL1α*	5.04E-06
*ITGβ1*	1.08E-03			*TGFβ1*	2.29E-02	*ITGβ1*		2.34E-03				*GlI3*	2.08E-03	*MMP16*	1.51E-03	*CSF3*	9.07E-03
*PTK2*	1.40E-02			*ELF3*	3.16E-05	*ESR1*		7.72E-05				*ICAM1*	2.13E-03			*ESR2*	5.90E-04
				*ITGβ1*	5.00E-03							*FGF9*	1.70E-02			*MMP16*	2.49E-03
												*IL1α*	4.71E-03				
												*CSF3*	6.27E-03				
												*ESR2*	2.46E-03				
												*MMP16*	1.15E-03				

VCaP Upregulated										VCaP Downregulated							
MousePre-osteoblast		Testosterone1nM		Estradiol10nM			Enzalutamide10µM		MousePre-osteoblast			Testosterone1nM		Estradiol10nM		Enzalutamide10µM	
Genes	P-value	Genes	P-value	Genes	P-value	Genes		P-value	Genes		P-value	Genes	P-value	Genes	P-value	Genes	P-value
*BMP2*	2.36E-03	*SOX9*	9.26E-06	*BMP2*	7.40E-05	*BMP7*		2.07E-02	*ICAM1*		2.32E-03	*BMP2*	4.29E-03	*ICAM1*	7.06E-03	*BMP6*	3.48E-05
*BMP6*	1.54E-03	*IGF1*	6.05E-05	*BMP6*	3.56E-02	*SHH*		2.13E-03	*FGF9*		1.83E-05	*BMP6*	2.28E-02	*FGF9*	6.10E-03	*IHH*	4.17E-04
*IHH*	3.87E-03			*BMP7*	2.73E-03	*SOX9*		4.39E-04	*MMP16*		2.40E-04	*BMP7*	2.34E-02	*MMP16*	1.82E-04	*GlI1*	9.31E-05
*SHH*	3.44E-03			*IHH*	4.27E-04	*PTCH1*		9.15E-03				*IHH*	6.40E-03			*ICAM1*	9.74E-06
*SOX9*	9.91E-04			*SHH*	3.80E-02	*GlI2*		1.36E-03				*SHH*	5.20E-03			*FGF9*	4.97E-04
*PTCH1*	2.64E-02			*SOX9*	1.63E-05	*IGF1*		1.75E-02				*PTCH1*	6.53E-03			*IL1α*	1.11E-03
				*GlI1*	9.62E-03	*TGFβ1*		2.23E-02				*GlI1*	2.34E-04			*MMP16*	3.03E-05
				*GlI2*	8.63E-03	*ITGβ1*		3.52E-02				*GlI2*	3.99E-04				
				*GlI3*	1.01E-02							*ICAM1*	5.63E-03				
				*IGF1*	1.81E-05							*FGF9*	2.71E-03				
												*IL1α*	1.29E-04				
												*MMP16*	1.25E-03				

Gene expression analysis was conducted using RT-qPCR. Statistical significance was ascertained by comparing the relative mRNA expression in 3D heterotypical organoids (treated with hormone agonist and anti-AR treatment) versus corresponding 3D cancer monocultures. Human specific primers used in the assay are summarized in Additional file 1: Table S1. Data represent mean ± SD, n = 3, *p < 0.05; ** p < 0.01; ***p < 0.001; ****p < 0.0001

### Hormone regulation of *TNC* expression

**Fig. 2:**
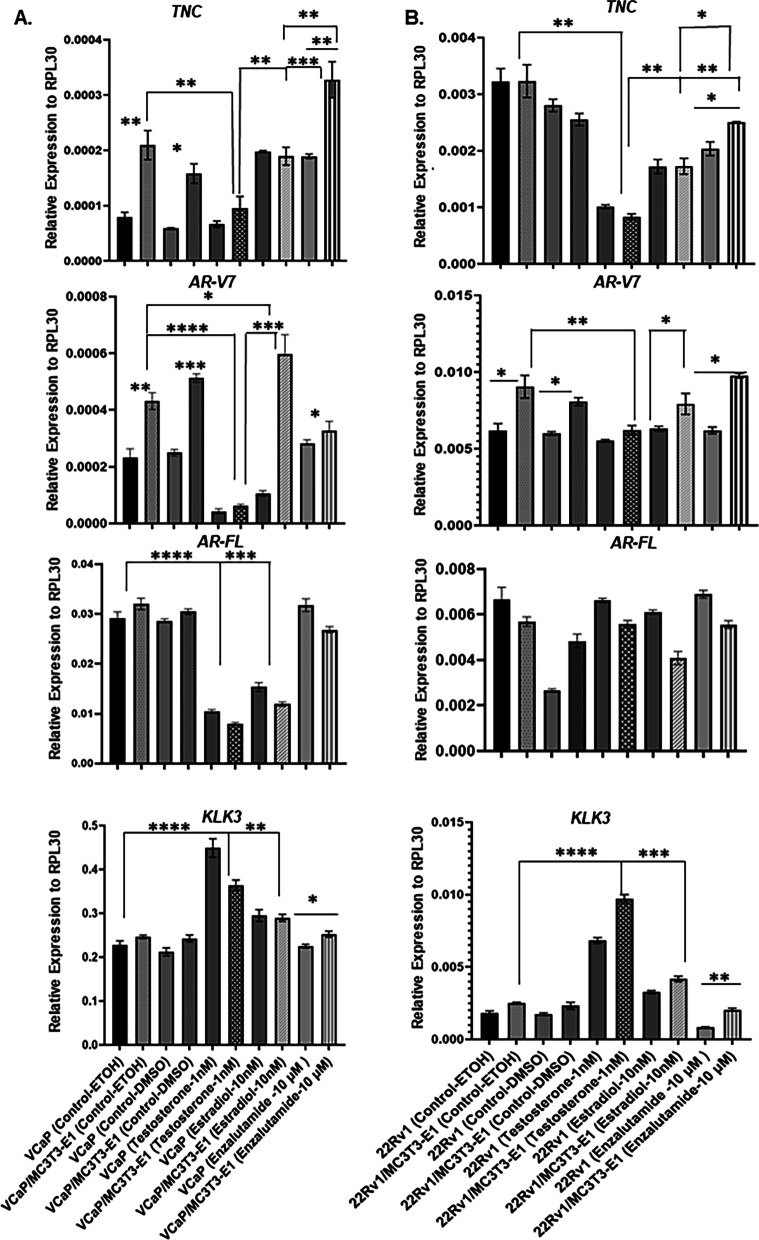
*TNC and AR-V7* expression decrease in 3D heterotypical organoids treated with testosterone. **A** and **B** Effect of testosterone (1 nM), estradiol (10 nM), and enzalutamide (10 µM) on *TNC, AR-V7, AR-FL,* and *Kallikrein related peptidase 3 (KLK3)/* Prostate Specific antigen (PSA) in 3D prostate cancer (VCaP or 22Rv1) and heterotypical (VCaP-22Rv1/MC3T3-E1) organoids using RT-qPCR. Human-specific primers were used for RT-qPCR to exclude transcripts from mouse derived preosteoblasts (MC3T3-E1). All data represent mean ± SD analyzed by unpaired students t-test (n = 3) *p < 0.05; ** p < 0.01; ***p < 0.001; ****p < 0.0001

Expression of *TNC* using human-specific primers was evaluated in heterotypical organoids. A significant increase in *TNC* expression occurred in VCaP in heterotypical organoids compared to corresponding 3D cancer monocultures (Fig. 2A). In 22Rv1, the basal expression of *TNC* was higher compared to VCaP and a *TNC* induction due to interaction with preosteoblasts was not evident (Fig. 2B). Both testosterone and estradiol induced *TNC* expression in preosteoblasts (Additional file 4: Fig. S2A, B). However, in organoid prostate cancer cells, testosterone significantly repressed *TNC* expression compared with vehicle and estradiol conditions. In contrast, enzalutamide significantly induced *TNC* expression in both VCaP and 22Rv1 in 3D heterotypical organoids compared to testosterone and estradiol treatment (Fig. 2A, B).

### Hormone regulation of *AR-V7* expression

**Fig. 3:**
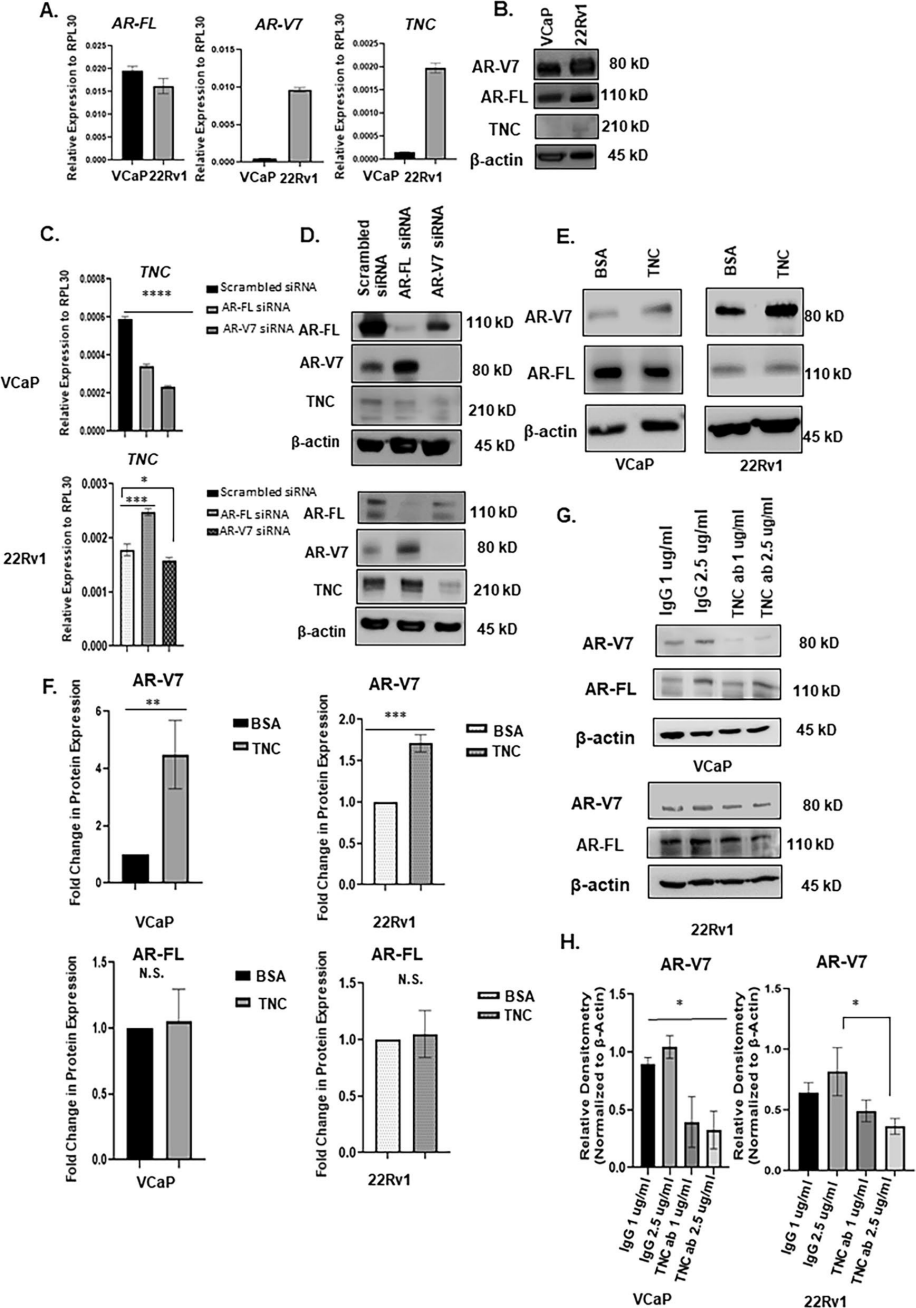
AR-V7 regulate *TNC* expression and TNC upregulate AR-V7 protein. **A** VCaP and 22Rv1 cells grown in 5% csFBS containing media were assessed for AR-FL, AR-V7, and TNC transcript expression using RT-qPCR and **B** protein level using Western blot. **C** VCaP and 22Rv1 treated with siRNA targeting AR-FL and AR-V7 to evaluate TNC transcript level using RT-qPCR and **D** protein level using Western blot analysis. **E** VCaP and 22Rv1 seeded on BSA versus TNC for 72 h in 5% csFBS containing media followed by Western blot analysis of AR-V7 and AR-FL protein expression with β-actin as loading control. **F** Densitometry quantification of AR-V7 and AR-FL protein expression depicted in (**E**) for n = 3 biological replicates. N.S. represents no significance. **G** VCaP and 22Rv1 treated with anti-tenascin monoclonal antibody (BC-24) at increasing concentrations (1, 2.5 μg/ml) and compared to Isotype/IgG control at concentrations (1, 2.5 μg/ml). AR-V7 and AR-FL protein levels were assessed using Western blots. **H**. Densitometric analysis of Western blot images of AR-V7 expression in VCaP and 22Rv1 treated with IgG or anti-tenascin monoclonal antibody (BC-24) for n = 3 biological replicates. All data represent mean ± SD analyzed by unpaired students t-test (n = 3) *p < 0.05; ** p < 0.01; ***p < 0.001; ****p < 0.0001

Basal constitutive expression of the AR-V7 variant using human-specific primers was detected in both VCaP and 22Rv1 cells at both the transcript (Fig. 2A, B, 3A) and protein level (Fig. 3B). However, interaction with preosteoblasts in heterotypical organoids significantly elevated *AR-V7* transcript expression in prostate cancer cells, while testosterone significantly repressed expression (Fig. 2A, 2B). In contrast, both estradiol and enzalutamide induced *AR-V7* expression in both VCaP and 22Rv1 in heterotypical organoids compared to corresponding cancer-only organoids. Interestingly, estradiol induction of *AR-V7* expression did not correlate with androgen receptor full length (*AR-FL*) expression as the latter was either significantly repressed in VCaP or not affected in 22Rv1 in heterotypical organoids (Fig. 2A, 2B).

### Reciprocal regulation of AR-V7 and TNC

Relative mRNA expression of *AR-V7* in basal (no hormone) conditions for 72 h revealed that the expression of therapeutic resistant variant was 32 fold higher in 22Rv1 compared to VCaP (Fig. 3A). Similarly, the expression of *TNC* was 18 fold higher in 22Rv1 compared to VCaP indicating a positive correlation between *AR-V7* and *TNC* (Fig. 3A). A similar trend was observed in the heterotypical organoid cultures (Fig. 2A and B). Western blot analyses confirmed TNC protein expression was higher in 22Rv1 compared to VCaP (Fig. 3B). The differential role of AR-FL and AR-V7 in gene regulation is known [32]; however, it is not yet understood if either AR-FL or AR-V7 transcriptionally regulates *TNC* expression in prostate cancer. To address this, we show that doxycycline inducible AR-V7 expression in LNCaP^AR−V7/pLenti^ induced *TNC* expression suggesting that AR-V7 transcriptionally regulate TNC expression (Additional file 5: Fig. S3B). In VCaP cells, a decrease in *TNC* expression by 50% was observed under AR-FL knockdown conditions while knockdown of AR-V7 resulted in downregulation of *TNC* by 70% (Fig. 3C). To validate, Western blot analyses revealed that knockdown of AR-V7 and not AR-FL abrogated TNC protein expression (Fig. 3D). Interestingly, in 22Rv1 cells, knock down of AR-FL stimulated both *AR-V7* and *TNC* expression whereas knockdown of AR-V7 significantly reduced TNC expression at both the transcript and protein level (Fig. 3C, 3D and Additional file 4: Fig. S2C). These results suggested that AR-V7 transcriptionally regulates *TNC* independent of AR-FL.

**Fig.4:**
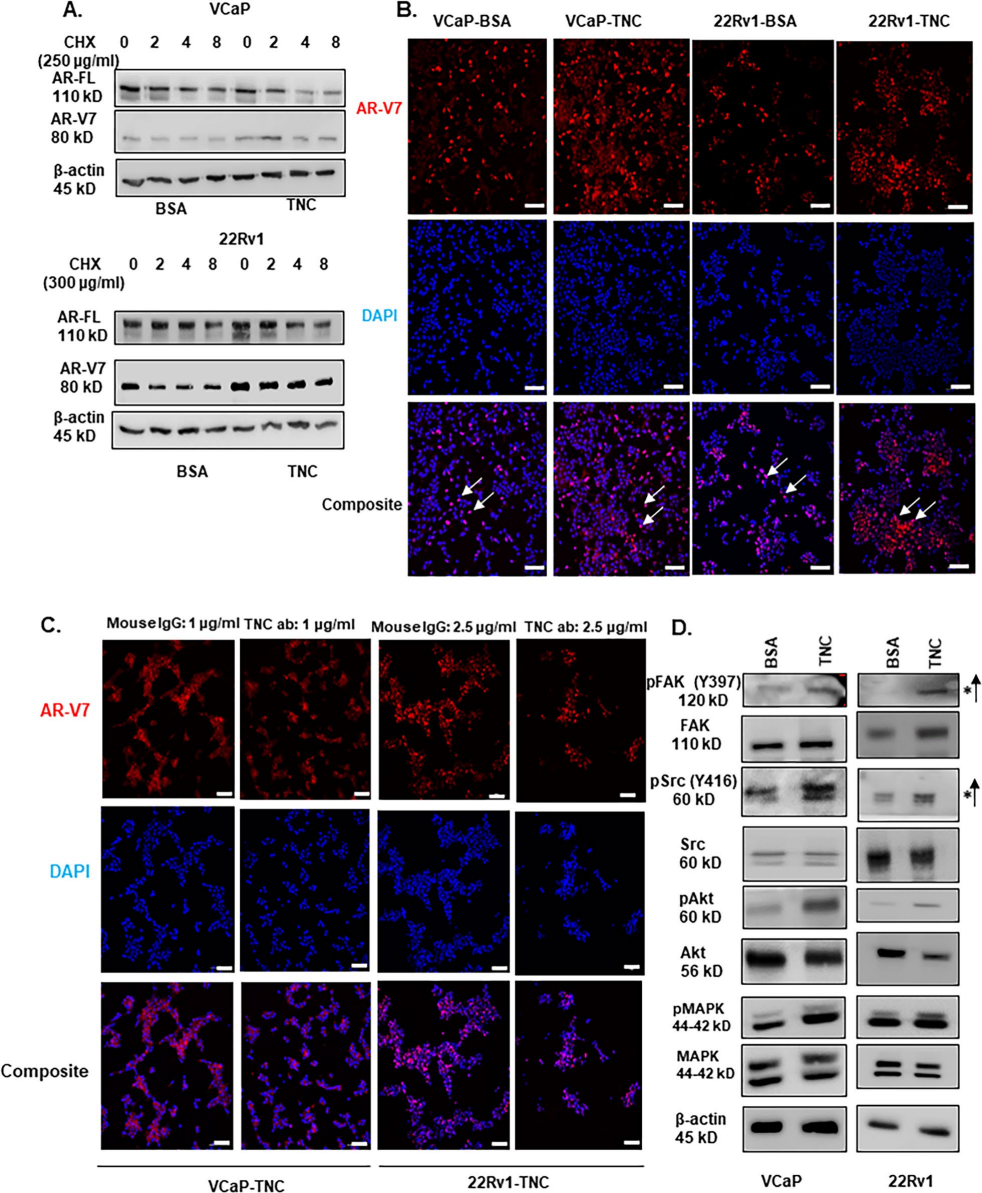
TNC induce post-translational stability of AR-V7 and activate FAK and Src signaling. **A** VCaP and 22Rv1 cultured on BSA versus TNC in 5% csFBS containing media were treated with cycloheximide (VCaP-250 μg/ml; 22Rv1-300 μg/ml) and Western blot conducted on protein lysate collected at 0,2,4, and 8 h. The cycloheximide chase experiment was conducted for n = 3 biological replicates for each cell line. **B** ICC of AR-V7 nuclear localization (white arrow) in both VCaP and 22Rv1 cultured on TNC compared to BSA coated IbiTreat chamber slides (Scale bar, 10 × 100 µm). **C** VCaP and 22Rv1 were cultured on TNC coated IbiTreat chamber slides followed by treatment with isotype control (IgG) or anti-tenascin monoclonal antibody (BC-24) at a concentration of 1 µg/ml (VCaP) and 2.5 µg/ml (22Rv1) respectively for 72 h. The nuclei are counterstained with DAPI. All ICC images represented in Fig. 4. were obtained using Nikon A1 confocal microscope (Scale bar, 10 × 100 µm) and repeated for n = 3 biological replicates. **D** Western blot analysis FAK, Src, Akt, and MAPK phosphorylation status in VCaP and 22Rv1 cultured on BSA versus TNC for n = 3 biological replicates

Next, the effect of extrinsic TNC in regulating AR-FL and AR-V7 in prostate cancer cells was evaluated. Transcript and protein levels of AR-FL and AR-V7 in VCaP and 22Rv1 cells, cultured on BSA or TNC coated wells in 5% csFBS conditions for 72 h were analyzed. There were no differences in relative mRNA expression of either *AR-FL* or *AR-V7* in both prostate cancer cell lines between BSA versus TNC conditions (Additional file 4: Fig. S2D). However, Western analyses showed the protein level of AR-V7 was significantly higher in both cell lines when cultured on TNC relative to BSA. In contrast, no change in AR-FL protein level was detected in TNC conditions (Fig. 3E, F). Cycloheximide chase assay followed by Western blot analysis confirmed that TNC-induced post-translational stability of AR-V7 (Fig. 4A). The relative levels of AR-V7 in both VCaP and 22Rv1 cells plated on BSA versus TNC treated with cycloheximide is depicted in Additional file 6: Fig. S4A. Further, treatment with TNC neutralizing antibody resulted in downregulation of AR-V7 protein level in both VCaP and 22Rv1 in a dose-dependent manner with no impact on AR-FL protein (Fig. 3G, H, and Additional 4: Fig. S2E). Interestingly, we also observed that autocrine *TNC* expression increased significantly in VCaP and 22Rv1 when plated on TNC-coated culture dishes (Additional file 4: Figure S2D). These data suggested a positive feedback regulation where TNC post-translationally stabilizes AR-V7 and the latter can transcriptionally regulates *TNC* expression in prostate cancer cells.

### TNC Induces an Increase in Nuclear AR-V7 and Activates FAK and Src Signaling.

TNC is observed to induce morphological and EMT-like changes in breast cancer cell lines [45]. Interestingly, we observed spheroid like morphology and loss of cell–cell contact-characteristic of epithelial cells in both VCaP and 22Rv1 when cultured on TNC (Additional file 7: Fig. S5C) Next, we evaluated the localization of AR-V7 in VCaP and 22Rv1 cells cultured on TNC. ICC revealed a significant increase in AR-V7 positive nuclei in both cell types cultured on TNC relative to BSA (Fig. 4B and Additional file 6: Fig. S4B and Additional file 7: Fig. S5A). To validate, treatment with TNC neutralizing antibody significantly reduced AR-V7 nuclear intensity (Fig. 4C and Additional file 6: Fig. S4C and Additional file 7: Fig. S5B).

**Fig. 5:**
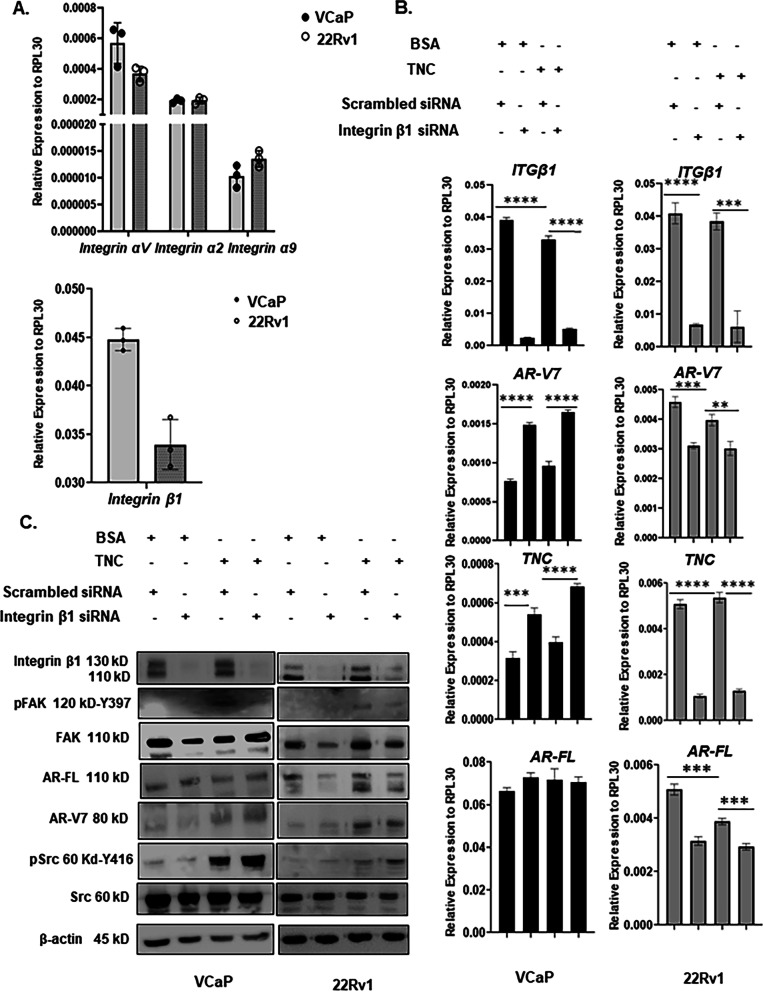
TNC-induced post-translational stability of AR-V7 is not mediated by Integrin mediated FAK activation. **A** RT-qPCR analysis reveal that VCaP and 22Rv1 express *integrin α*v/2/9 and *β*1. **B** RT-qPCR analysis on expression of *AR-V7*, *TNC,* and *AR-FL* in VCaP and 22Rv1 cultured on BSA or TNC after integrin β1 knockdown using siRNA **C** Western blot analysis on the impact of integrin β1 knockdown on TNC signaling in VCaP and 22Rv1. SiRNA knockdown of integrin β1 impedes TNC-induced FAK activation but does not affect Src activation nor AR-V7 post-translational stability. All data represent mean ± SD analyzed by unpaired students t-test (n = 3); ** p < 0.01; ***p < 0.001; ****p < 0.0001

To evaluate signaling pathways, we compared TNC-induced activation of kinases associated with cell adhesion and survival such as FAK, Src, Akt, and MAPK. In both VCaP and 22Rv1, TNC significantly induced activation of FAK as indicated by an increase in its auto-phosphorylation status at Y397 which is indicative of integrin clustering. In addition, TNC activated Src kinase as indicated by increase in its phosphorylation status at Y416 (Fig. 4D and Additional file 7: Fig. S5D). Concurrently, we observed that TNC also induced an increase in phosphorylation of Akt and MAPK-both downstream effectors of FAK and Src activation (Fig. 4D). These results suggest that TNC induces an increase in nuclear localized AR-V7 and activates FAK and Src mediated signaling in prostate cancer cells.

### TNC -induced AR-V7 Stability is Dependent on Src Activation

Integrin heterodimers αv/2/7/8/9-β1 and αv-(β)3/6 are known to bind to TNC and induce intracellular signaling [16]. We observed that both VCaP and 22Rv1 express *integrins αv/2/9* and *integrin β1* (Fig. 5A). *Integrin β3* expression was very low in both VCaP and 22Rv1 and *integrin β6* was not detected in either cell lines (data not shown). This suggests that integrin β1 is the major hetero-dimerization partner for integrin αv/2/9 that can bind to TNC. To understand if TNC-induced AR-V7 post-translational stability is mediated via integrin signaling, integrin β1 was knocked down to disrupt formation of integrin heterodimers. Interestingly, knockdown of integrin β1 induced *AR-V7* and *TNC* expression with no effect on *AR-FL* expression in VCaP while in 22Rv1, the knockdown resulted in suppression of *AR-FL*, *AR-V7*, and *TNC* expression (Fig. 5B). Corresponding Western blot analysis showed that knockdown of integrin β1 impeded TNC-induced FAK activation as evidenced by the reduction of auto-phosphorylation at Y397 (Fig. 5C). Contrary to differential regulation of either *AR-FL* or *AR-V7* transcript expression between cell lines, knockdown of integrin β1 did not have an impact on TNC-induced Src activation nor AR-V7 protein stability in either VCaP or 22Rv1 (Fig. 5C). These results suggested the following: firstly, integrin β1 regulates AR-V7 splicing but not its post-translational protein stability, and secondly, TNC can induce Src activation independent of integrin mediated FAK activation.

**Fig. 6:**
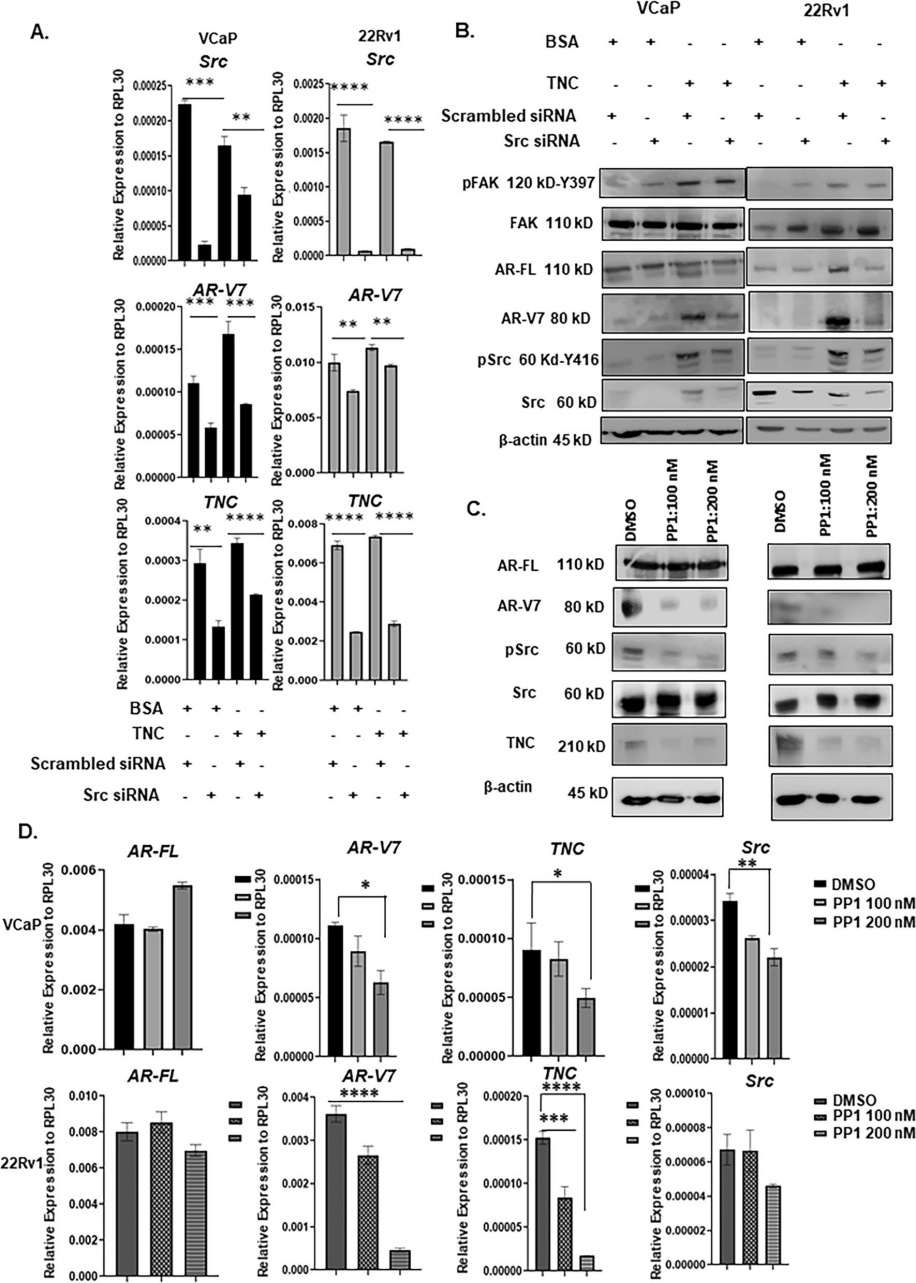
Activated Src regulates post-translational stability and alternative splicing of AR-V7. **A** RT-qPCR analysis on the impact of Src knockdown using siRNA on *AR-V7* and *TNC* expression in both VCaP and 22Rv1 cultured on BSA or TNC. **B** Western blot analysis on Src knockdown in VCaP and 22Rv1 cultured on BSA and TNC. siRNA knockdown of Src does not affect TNC-induced FAK activation but destabilizes AR-V7. **C** Western blot analysis of Src kinase inhibition using PP1with DMSO as the vehicle control on AR-V7, AR-FL, and TNC protein level. **D** RT-qPCR analysis on *AR-FL*, *AR-V7*, and *TNC* expression followed by Src kinase inhibition using PP1 inhibitor All data represent mean ± SD analyzed by unpaired students t-test (n = 3) *p‹0.05, ** p < 0.01, ***p < 0.001, ****p < 0.0001

**Fig. 7:**
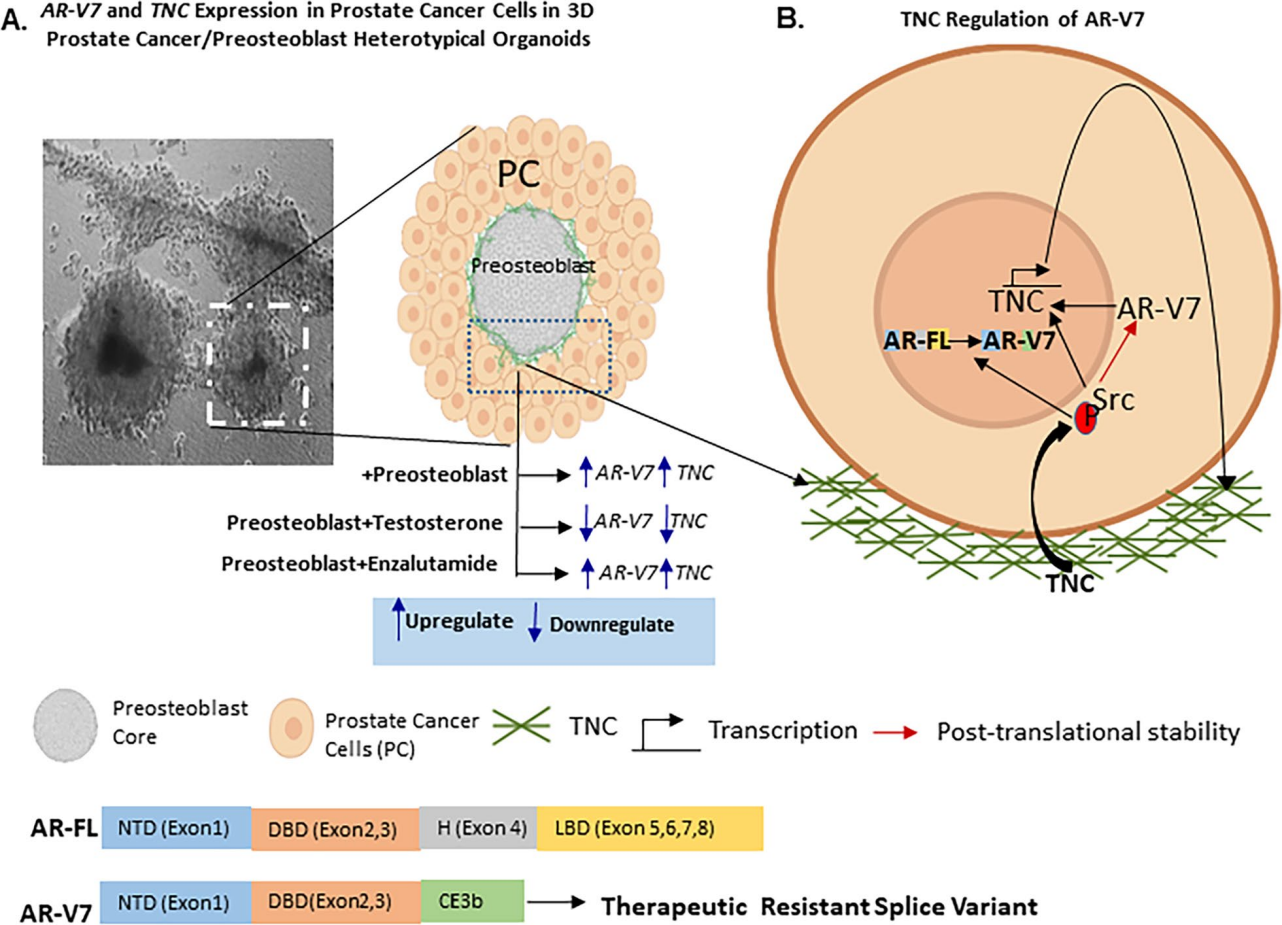
Model of regulation of AR-V7 in prostate cancer cells interacting with Preosteoblast and TNC. **A** Prostate cancer cells interact with preosteoblast forming 3D heterotypical organoids. The preosteoblast organizes to form the inner core and prostate cancer cells compartmentalize around the preosteoblast. TNC is secreted by preosteoblast onto its surface in regions of contact with prostate cancer cells. Both *TNC* and *AR-V7* transcript levels are upregulated in prostate cancer cells interacting with preosteoblast, which is further induced by Enzalutamide (Enz) and suppressed by Testosterone (Test). The 3D heterotypical organoid depicted is composed of VCaP and mouse preosteoblasts-MC3T3-E1. The live cell image of the 3D heterotypical organoids was captured using 20 × objective. **B** TNC induce Src activation in prostate cancer cells regulating AR-V7 splicing and stability. This suggests a positive feedback effect where the osteogenic microenvironment components like preosteoblasts and TNC can select for AR-V7 in prostate cancer cells via Src activation. Reciprocally both AR-V7 and activated Src regulate autocrine TNC expression in prostate cancer cells further modifying the osteogenic microenvironment. The image was created with Biorender.com

High content screening has shown that Src family of kinases regulates AR-V7 expression in CRPC [46]. Accordingly, Src was knocked down in both VCaP and 22Rv1 to determine if TNC-induced AR-V7 protein stability is mediated via Src activation. As shown in Fig. 6B**,** Src knockdown downregulated AR-V7 at the protein level in both VCaP and 22Rv1 cultured on TNC, thus implicating Src as a critical mediator of TNC-induced AR-V7 post-translational stability. Interestingly, knockdown of Src suppressed *TNC* and *AR-V7* expression in both VCaP and 22Rv1 (Fig. 6A). However, Src knockdown did not affect *AR-FL* expression in VCaP while the latter was suppressed in 22Rv1 (Additional file 8: Fig. S6A). These results suggest that Src regulation of *AR-V7* splicing is not necessarily dependent on *AR-FL* expression. We wanted to validate via inhibition of Src kinase activity to assess the impact on alternative splicing of *AR-V7*. Src kinase inhibition using small reversible molecule inhibitor-PP1 resulted in a suppressed expression of both AR-V7 and TNC transcript and in protein expression in a dose-dependent manner in both VCaP and 22Rv1. There was, however, no change on *AR-FL* transcript or protein expression (Fig. 6C, D). These data suggest that the kinase activity of Src is critical for regulating *TNC* expression, alternative splicing and post-translational stability of AR-V7 specifically, independent of AR-FL. In summary, the results reported here suggest a positive feedback effect where preosteoblast and TNC in the osteogenic microenvironment regulate ligand independent AR-V7 splicing and stability via Src activation, while reciprocally, activated Src and AR-V7 regulate autocrine *TNC* expression in prostate cancer cells further modifying the osteogenic microenvironment (Fig. 7).

## Discussion

Majority of prostate cancer patients with advanced disease progress with metastases to the bone. This indicates an exquisite tropism by disseminated prostate cancer cells to both survive and proliferate in the bone microenvironment [41]. In the bone, prostate cancer foci are observed both in the bone marrow and directly on trabecular bone surface interacting with the osteogenic niche on sites with elevated deposits of TNC [24, 47]. In this study, utilizing novel prostate cancer/preosteoblast heterotypical organoids, we demonstrate that TNC is likely a key regulatory component of prostate cancer-preosteoblast interactions. In addition, data reported here suggests that TNC may play a key role in regulating therapeutic resistance in prostate cancer via post-translationally stabilizing AR-V7.

AR-V7 is the molecular marker for resistance in patients with bone metastatic CRPC [34, 49, 50]. We report here that interaction with preosteoblasts significantly upregulate *AR-V7* transcript expression in prostate cancer cells. It is established that estrogen, bioavailable as 17 β-estradiol via activity of aromatase in males, have a gender-neutral role in regulating bone homeostasis [44]. Our data suggests that the osteogenic niche, together with either estradiol or enzalutamide may upregulate *AR-V7* splicing in disseminated prostate cancer cells independent of testosterone action. *TNC* expression in prostate cancer cells in heterotypical organoids is regulated similar to *AR-V7*, where testosterone suppressed gene expression compared to estradiol or enzalutamide. The data presented here suggest that AR-V7 transcriptionally regulates *TNC* expression in prostate cancer cells and TNC reciprocally induces AR-V7 protein stability and nuclear localization. This suggests that TNC expression in an osteogenic niche may be selective for CRPC evolution by regulating both alternative splicing and selectively stabilizing AR-V7 protein in disseminated prostate cancer cells, and AR-V7 positive cancer cells further modulates the tumor niche by regulating autocrine *TNC* expression. Further, we report here that one of the mechanisms through which TNC and the bone niche regulate AR-V7 is likely via Src activation.

In prostate cancer, Src activation has been associated with CRPC, metastatic fitness, and its highest activity is observed in prostate bone metastases [51, 52]. In addition to TNC, Src can be activated by other factors that are expressed in the bone microenvironment including growth factors such as IGF, Hedgehog signaling, bone effectors such as BMPs, TGFβ, integrin mediated signaling etc. [52, 53]. Gene expression analysis presented in this study indicate that these upstream regulators of Src, including TNC, are induced in prostate cancer cells interacting with preosteoblasts. Importantly, the finding that these genes are suppressed by testosterone, and induced by both enzalutamide and estradiol suggests that prostate cancer interaction with the osteogenic niche in therapeutic conditions produces a microenvironment for hormone independent activation of Src. It would follow that activated Src functions to promote splicing and protein stability of AR-V7, and up-regulates *TNC* expression in disseminated prostate cancer cells within the bone microenvironment. Intriguingly, Src activation is also associated with survival and development of breast cancer metastases in the bone [54, 55]. Like prostate, ER + breast cancer also predominantly metastasize to the bone [56]. It is possible that Src activation could be a common and critical mechanism associated with competence of prostate and breast cancer to survive and progress to therapeutic resistance in the bone microenvironment.

Expression of AR variants, namely AR-V7, is not cancer specific as it is detected in several human tissues including liver, spleen, placenta, brain, small intestine, and white blood cells indicating that AR variants likely have a physiological function which is currently unknown [57, 58]. AR-FL plays a crucial role in cutaneous wound healing [59]. Coincidentally, our finding that AR-V7 regulates wound repair associated ECM protein like TNC raises an intriguing possibility that the splice variant has a role in the wound repair process. Apart from TNC, AR-V7 in CRPC regulates other repair centric genes, including BMP7, Gli3, HIF-2α, SLC3A2, etc. [60]. These genes play a major role in wound repair by inducing neurite outgrowth, immune inactivation, angiogenesis, response to nutrient stress, and modulation of cell adhesions, all processes that are pro-tumorigenic in cancer condition [60–64]. The concept coined by Dvorak that tumors are “wounds that do not heal,” [65] raises the question as to whether AR-V7 functions in non-pathological condition is to regulate an **emergency or emergent tissue system biology** to effect rapid repair and establishment of homeostasis. It follows then in pathological condition like prostate cancer, AR-V7 is selected to restore AR signaling in androgen depleted conditions, which effectively transforms the tumor microenvironment to an emergent/chronic wound repair status by regulating expression of genes like TNC resulting in hormone independent growth, survival, and progression [65].

## Conclusions

To summarize, the present study addresses the complex interaction between prostate tumor and microenvironment components in regulating expression of therapeutic resistant variant like AR-V7. According to Paget’s “seed and soil” hypothesis, TNC in the reactive endosteum is a fertile soil that can facilitates prostate cancer (seed) colonization and growth on trabeculae bone [24]. Herein, we identify that TNC also post-translationally stabilize AR-V7, independent of testosterone regulation, via Src activation in prostate cancer cells. These data implicate the reactive microenvironment as crucial in facilitating therapeutic resistance. Consistent with these findings, TNC expression is also associated with resistance to tamoxifen as first line of treatment in patients with metastatic breast cancer [66]. Thus, these results raises the possibility that the TNC positive osteogenic niche can regulate and select the survival of cancer cells that are resistant to AR or estrogen receptor targeted treatments. Therefore, both TNC and its downstream effector, Src, may be potentially used as criterion for consideration for novel treatment strategies for prostate and breast bone metastases, or can serve as dual targets for therapeutic approach.

## Supplementary Information

**Additional file 1**: **Table S1. **Human-Specific Primer Sequences for Genes

**Additional file 2**: **Table S2.** Antibody List

**Additional file 3**: **Fig. S1.** Testosterone and estradiol regulate genes involved in osteogenesis. A Heat map representing the differentially regulated genes involved in osteogenesis by testosterone (1 nM) and estradiol (10 nM) in mouse preosteoblast (MC3T3-E1) using RT2 Osteogenesis PCR array.

**Additional file 4**: **Fig. S2.** Gene expression analysis. A and B The effect of testosterone (1 nM) and estradiol (10 nM) on TNC expression in MC3T3-E1 confirmed by RT-qPCR and Western blot analysis. C RT-qPCR analysis of VCaP and 22Rv1 seeded on 6 well plate treated with siRNA targeting AR-FL and AR-V7. D RT-qPCR analysis of AR-FL, AR-V7, and TNC expression in VCaP and 22Rv1 plated on BSA versus TNC. N.S. represents no significance. E. Densitometric analysis of Western blot images of AR-FL expression in VCaP and 22Rv1 treated with IgG or anti-tenascin monoclonal antibody for n=3 biological replicates. Data represent mean ± SD, n=3, N.S.: Not Significant, *p<0.05, ** p<0.01 ***p<0.001, ****p<0.0001.

**Additional file 5**: **Fig. S3.** Doxycycline induced AR-V7 expression in LNCaPAR-V7/pLenti . A Doxycycline inducible AR-V7 expression in LNCaPAR-V7/pLenti verified by Western blot B RT-qPCR analysis of AR-FL, AR-V7, and TNC expression in doxycycline induced LNCaPAR-V7/pLenti cell line. C ICC images of AR-V7 in LNCaPAR-V7/pLenti cells treated with Dox (2.5 ng/µl). Nuclei counterstained with DAPI (Scale bars, 10x: 100µm). Data represent mean ± SD, n=3, * p‹0.05, ** p<0.01 ***p<0.001, ****p<0.0001.

**Additional file 6**: **Fig. S4.** TNC modulates AR-V7 protein stability. A VCaP and 22Rv1 plated on BSA versus TNC in 5% csFBS containing media was treated with cycloheximide. AR-V7 band intensity was normalized to β-actin and then normalized to time=0 hr (representative prior to treatment). Data represent mean ±SD for n=3 biological replicates. B. ICC of AR-V7 nuclear localization (white arrow) in both 22Rv1 and VCaP cultured on TNC compared to BSA coated IbiTreat chamber slides. The nuclei are counterstained with DAPI. All ICC images were obtained using Nikon A1 confocal microscope (Scale bar, 20x 50µm; 40x 20 µm). C. 22Rv1 and VCaP were cultured on TNC coated IbiTreat chamber slides followed by treatment with isotype control (IgG) or anti-tenascin monoclonal antibody at a concentration of 2.5 µg/ml (22Rv1) and 1 µg/ml (VCaP) respectively for 72 hours. The nuclei are counterstained with DAPI. All ICC images were obtained using Nikon A1 confocal microscope (Scale bar, 20x 50µm; 40x 20 µm).

**Additional file 7**: **Fig. S5.** TNC-induced increase in AR-V7 nuclear staining. A Fiji Image J quantification of AR-V7 positive nuclei in VCaP and 22Rv1 seeded on BSA versus TNC ( Scale bars, 10x: 100µm). B Fiji Image J quantification of AR-V7 nuclear intensity in VCaP and 22Rv1 seeded on BSA versus TNC (Scale bars, 10x: 100µm). C Live cell imaging of VCaP and 22Rv1 morphology seeded on BSA versus TNC using 20 x objective. D Densitometric analysis of Western blot images depicting pFAK and pSrc activation in VCaP and 22Rv1 seeded on BSA versus TNC for n=3 biological replicates. Data represent mean ± SD, n=3,*p<0.05, ** p<0.01

**Additional file 8**: **Fig. S6.** AR-FL expression with Src knockdown. A RT-qPCR following Src knockdown in VCaP and 22Rv1 plated on BSA versus TNC. N.S represents no significances. Data represent mean ± SD, n=3, ***p<0.001.

## Data Availability

All data generated and analyzed for this study are included in this article and the additional supplementary files.

## Abbreviations

TNC: Tenascin-C
AR: Androgen receptor
AR-V7: Androgen receptor variant 7
CRPC: Castration resistant prostate cancer
