# Supplementary material for: Androgen receptor variant-7 regulation by tenascin-c induced src activation
Source: Cell Commun Signal. 2022 Aug 10;20:119. doi: 10.1186/s12964-022-00925-0 (PMC9364530; doi:10.1186/s12964-022-00925-0)
Supplement: Supplementary file 2 — Additional file 1: Table S1. Human-Specific Primer Sequences for Genes [file 12964_2022_925_MOESM2_ESM.docx]

**Supplementary Table 1: Human-Specific Primer Sequences for Genes**

| *ARFL*  *Androgen Receptor - Full Length* | *F: CTCTAGCCTCAATGAACTGGG* | *IGF1*  *Insulin Growth Factor 1* | *F:CTAAGGAGGCTGGAGATGTATTG* |
| --- | --- | --- | --- |
|  | *R: CAGGAGTACTGAATGACAGCC* |  | *R: GGCTGATACTTCTGGGTCTTG* |
| *ARV7*  *Androgen Receptor - variant 7* | *F: TCAAAAGAGCCGCTGAAGGG* | *IHH*  *Indian Hedgehog* | *F: TGGGTGTATTACGAGTCAAAGG* |
|  | *R: CAACCCGGAATTTTTCTCCCAG* |  | *R: CCTCACGGCTGACAAGG* |
| *BMP2*  *Bone Morphogenetic Protein 2* | *F: CTATCAGGACATGGTTGTGGAG* | *IL1a*  *Interleukin 1-alpha* | *F: CCACCCTCTATCACTGACTTTC* |
|  | *R: GGGAAATATTAAAGTGTCAACTGGG* |  | *R: GAACTGTCAACACTGCACAAG* |
| *BMP6*  *Bone Morphogenetic Protein 6* | *F: GTTGTAAGAGCTTGTGGATGC* | *ITGB1*  *Integrin beta-1* | *F: TCATGACAGAAGGGAGTTTGC* |
|  | *R: TTCAAAGTCTCATCGTCCCAC* |  | *R: ACGGGCAGTACTCATTTTCC* |
| *BMP7*  *Bone Morphogenetic Protein 7* | *F: TTCGACAATGAGACGTTCCG* | *KLK3*  *Kallikrein Related Peptidase 3* | *F: ACCTGCACCCGGAGAGCT* |
|  | *R: TGGCTGTGATGTCAAACACC* |  | *R: TCACGGACAGGGTGAGGAAG* |
| *CSF3*  *Colony Stimulating Factor 3* | *F: CATAGCGGCCTTTTCCTCTAC* | *MMP16*  *Matrix Metallopeptidase 16* | *F: GCGACGGGAATTTTGTGTTC* |
|  | *R: CCATTCCCAGTTCTTCCATCT* |  | *R: GGCTGAATCAATACCATGAGGG* |
| *ELF3*  *E74-like ETS Transcription Factor 3* | *F: TCTATTTAGAGCCGGGTAGGG* | *PTCH1*  *Patched 1* | *F:* *ACTCCCAAGCAAATGTACGAG* |
|  | *R: TGCTAATCTCACAGGTTGCAG* |  | *R:  TTGAGTGGAGTTCTGTGCG* |
| *ESR1*  *Estrogen Receptor 1* | *F: GGGCTCTACTTCATCGCATTC* | *PTK2*  *Protein Tyrosine Kinase 2* | *F: GGAGTATGTCCCTATGGTGAAG* |
|  | *R: AGGGATTATCTGAACCGTGTG* |  | *R: CCCAGGTCAGAGTTCAATAGC* |
| *ESR2*  *Estrogen Receptor 2* | *F: GCAGAGGACAGTAAAAGCAAAG* | *RPL30*  *60S Ribosomal Protein L30* | *F: GAAGACGAAAAAGTCGCTGGA* |
|  | *R: AGCAGAAAGATGAAGCCCAG* |  | *R: TGGGCAGTTGTTAGCGAGAA* |
| *FGF9*  *Fibroblast Growth Factor 9* | *F: CGCCTAATATCTCCTGGGTTG* | *SHH*  *Sonic Hedgehog* | *F: CTACGAGTCCAAGGCACATATC* |
|  | *R: TCCTGCACACCGAAATAGTTC* |  | *R: CAGGTCCTTCACCAGCTTG* |
| *GLI1*  *Zinc Finger Protein GLI1* | *F: TGCAGCAGGAATTTGACTC* | *SOX9*  *SRY-Box Transcription Factor 9* | *F: CACGCTCACTCGACCTTG* |
|  | *R: ATGTACTGGGCTTTGAAGGG* |  | *R: ACACAAATGTCCAAAGGGAATTC* |
| *GLI2*  *Zinc Finger Protein GLI2* | *F: GGAGAGCAAGTTCCTGAACAT* | *TGFB1*  *Transforming Growth Factor beta-1* | *F: TTGATGTCACCGGAGTTGTG* |
|  | *R: ACCCCTCAAGCCTATTTAACAC* |  | *R: GTAGTGAACCCGTTGATGTCC* |
| *GLI3*  *Zinc Finger Protein GLI3* | *F: CAGCCCCTATGCAGACATTAT* | *TNC*  *Tenascin-C* | *F: AAAGGAAGGCGATCCCAGAC* |
|  | *R: TGGACAGTGTACGTTTTCGG* |  | *R: GGGTGTACCTCCTGTAATGGG* |
| *ICAM1*  *Intracellular Cell Adhesion Molecule 1* | *F: GGAGCTTCGTGTCCTGTATG* |  |  |
|  | *R: TGCCATCCTTTAGACACTTGAG* |  |  |
