# Supplementary material for: Androgen receptor variant-7 regulation by tenascin-c induced src activation
Source: Cell Commun Signal. 2022 Aug 10;20:119. doi: 10.1186/s12964-022-00925-0 (PMC9364530; doi:10.1186/s12964-022-00925-0)
Supplement: Supplementary file 3 — Additional file 2: Table S2. Antibody List [file 12964_2022_925_MOESM3_ESM.docx]

| **Antibody** | **Vendor** | **Cat. #** | **Dilution** |
| --- | --- | --- | --- |
| **Antibody Used for Treatment** | | | |
| mTNC-BC24 | Sigma | SAB4200782 | 0, 1, 2.5 μg/ml |
| mIgG Isotype Control | CST | #5415 | 0, 1, 2.5 μg/ml |
| **Western Blot** | | | |
| TNC | Sigma | AB19011 | 1:2000 |
| AR | CST | #5153 | 1:5000 |
| AR-V7 | CST | #19672 | 1:2000 |
| phospho-FAK | CST | #8556 | 1:1000 |
| FAK | CST | #3285 | 1:3000 |
| phospho-Src | CST | #6943 | 1:3000 |
| Src | CST | #2109 | 1:3000 |
| phospho-MAPK | CST | #4370 | 1:5000 |
| MAPK | CST | #9102 | 1:5000 |
| phospho-Akt | CST | #4060 | 1:2000 |
| Akt | CST | #9272 | 1:2000 |
| Integrin β1 | CST | #4706 | 1:3000 |
| β-Actin | CST | #3700 | 1:10000 |
| HRP-conjugated anti-mouse IgG | CST | #7076 | 1:5000 |
| HRP-conjugated anti-rabbit IgG | CST | #7074 | 1:2000 |
| **ICC** | | | |
| TNC (FITC-conjugated in house) | Sigma | AB19011 | 1:250 |
| AR-V7 | CST | #19672 | 1:500 (22Rv1)  1:100 (VCaP) |
| Alexa Fluor 594 goat anti-rabbit IgG | Fisher | A-11072 | 1:500 |

**Supplementary Table 2: Antibody List**
