# Supplementary figures and images for: Androgen receptor variant-7 regulation by tenascin-c induced src activation
Source: Cell Commun Signal. 2022 Aug 10;20:119. doi: 10.1186/s12964-022-00925-0 (PMC9364530; doi:10.1186/s12964-022-00925-0)

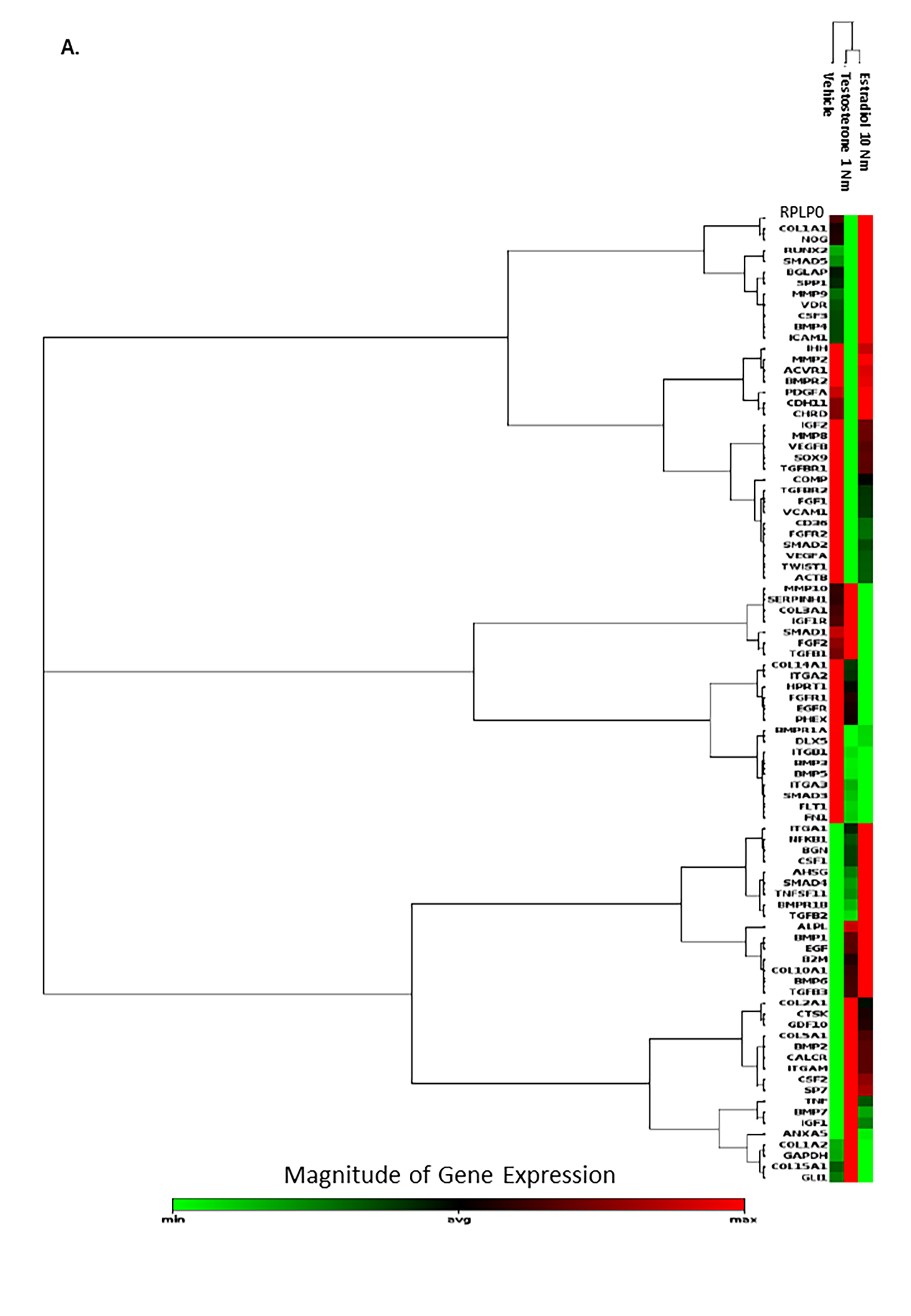

Supplement: Supplementary file 4 — Additional file 3: Fig. S1. Testosterone and estradiol regulate genes involved in osteogenesis. A Heat map representing the differentially regulated genes involved in osteogenesis by testosterone (1 nM) and estradiol (10 nM) in mouse preosteoblast (MC3T3-E1) using RT2 Osteogenesis PCR array. [file 12964_2022_925_MOESM4_ESM.tif]

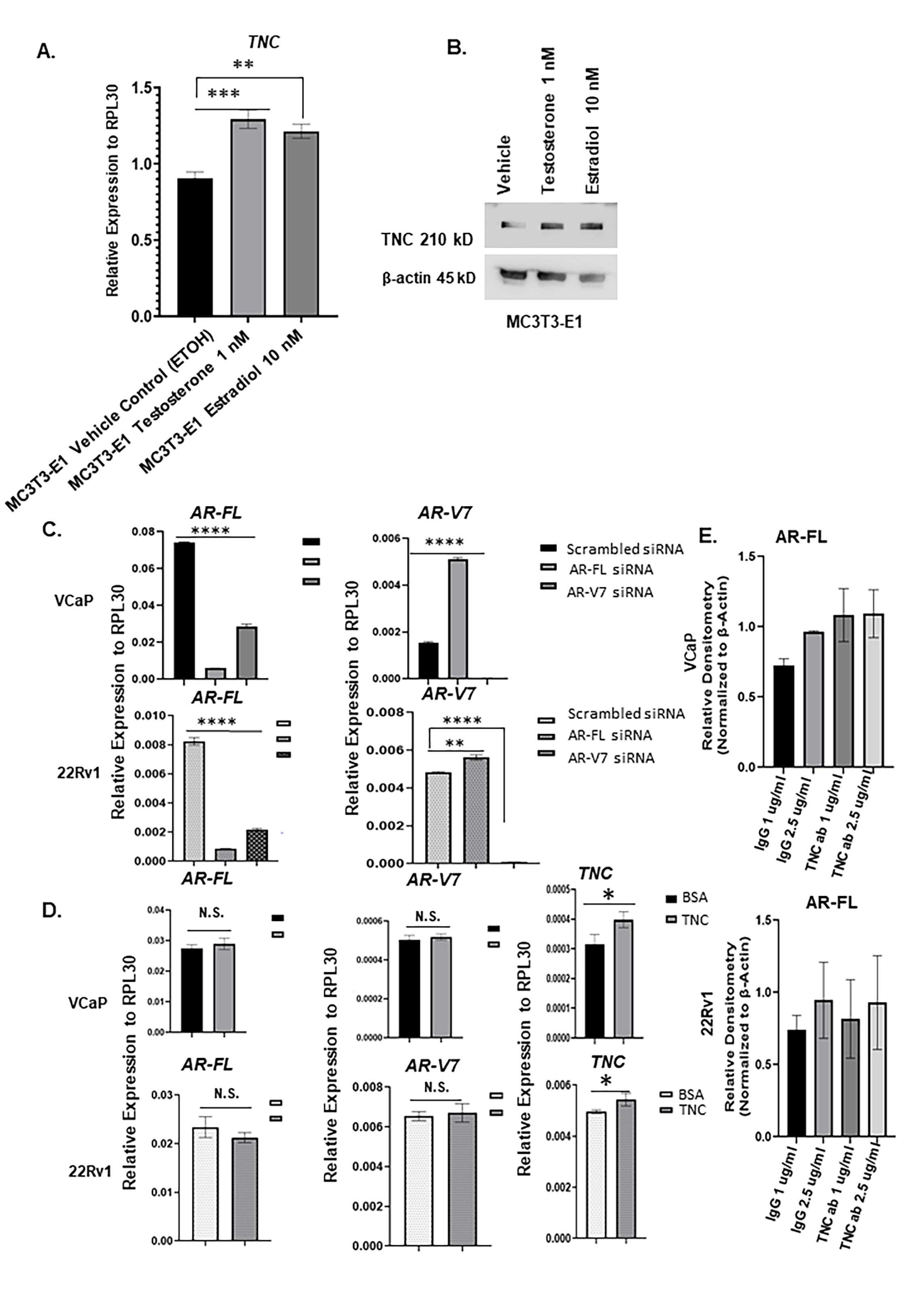

Supplement: Supplementary file 5 — Additional file 4: Fig. S2. Gene expression analysis. A and B The effect of testosterone (1 nM) and estradiol (10 nM) on TNC expression in MC3T3-E1 confirmed by RT-qPCR and Western blot analysis. C RT-qPCR analysis of VCaP and 22Rv1 seeded on 6 well plate treated with siRNA targeting AR-FL and AR-V7. D RT-qPCR analysis of AR-FL, AR-V7, and TNC expression in VCaP and 22Rv1 plated on BSA versus TNC. N.S. represents no significance. E. Densitometric analysis of Western blot images of AR-FL expression in VCaP and 22Rv1 treated with IgG or anti-tenascin monoclonal antibody for n=3 biological replicates. Data represent mean ± SD, n=3, N.S.: Not Significant, *p<0.05, ** p<0.01 ***p<0.001, ****p<0.0001. [file 12964_2022_925_MOESM5_ESM.tif]

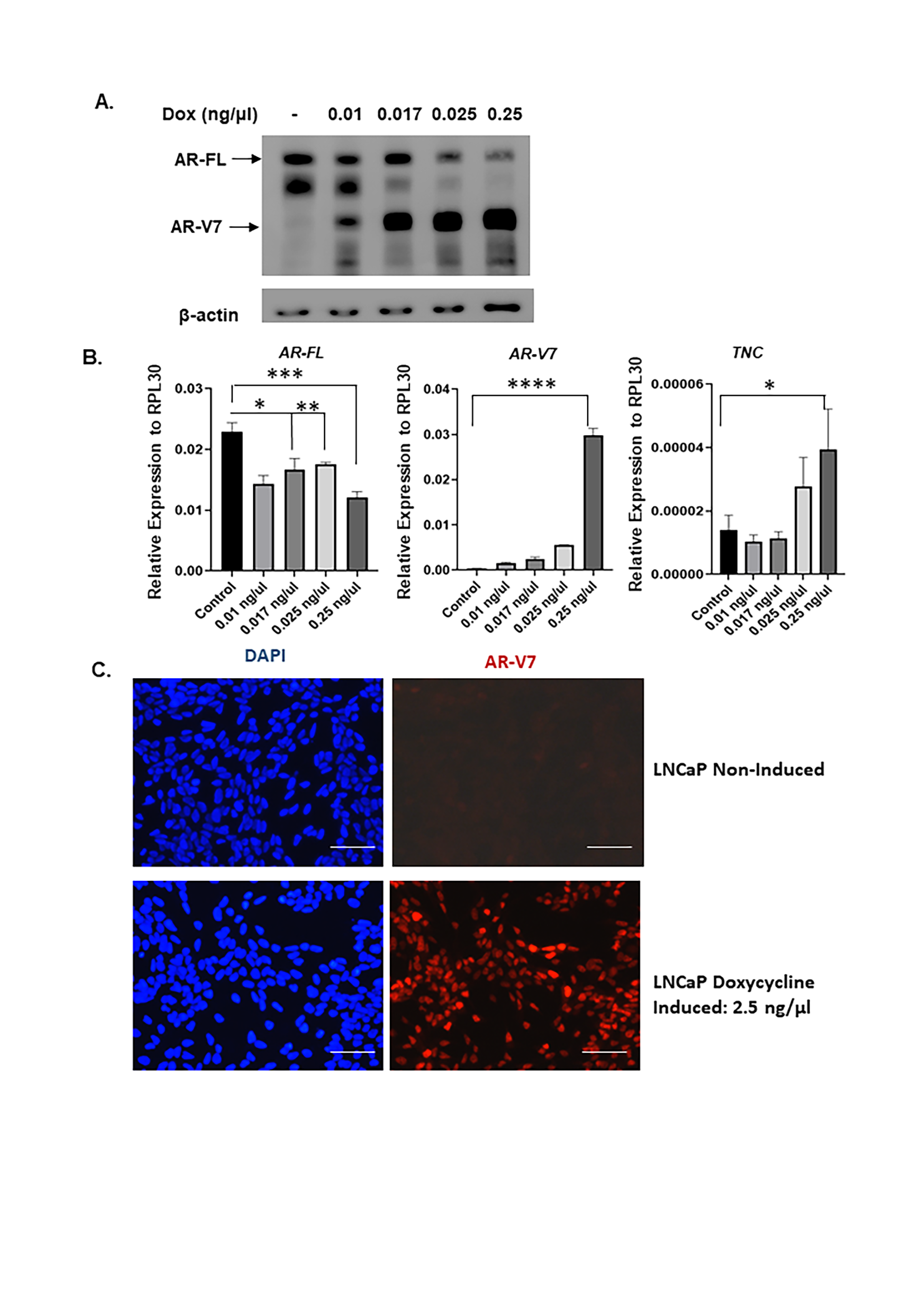

Supplement: Supplementary file 6 — Additional file 5: Fig. S3. Doxycycline induced AR-V7 expression in LNCaPAR-V7/pLenti . A Doxycycline inducible AR-V7 expression in LNCaPAR-V7/pLenti verified by Western blot B RT-qPCR analysis of AR-FL, AR-V7, and TNC expression in doxycycline induced LNCaPAR-V7/pLenti cell line. C ICC images of AR-V7 in LNCaPAR-V7/pLenti cells treated with Dox (2.5 ng/µl). Nuclei counterstained with DAPI (Scale bars, 10x: 100µm). Data represent mean ± SD, n=3, * p‹0.05, ** p<0.01 ***p<0.001, ****p<0.0001. [file 12964_2022_925_MOESM6_ESM.tif]

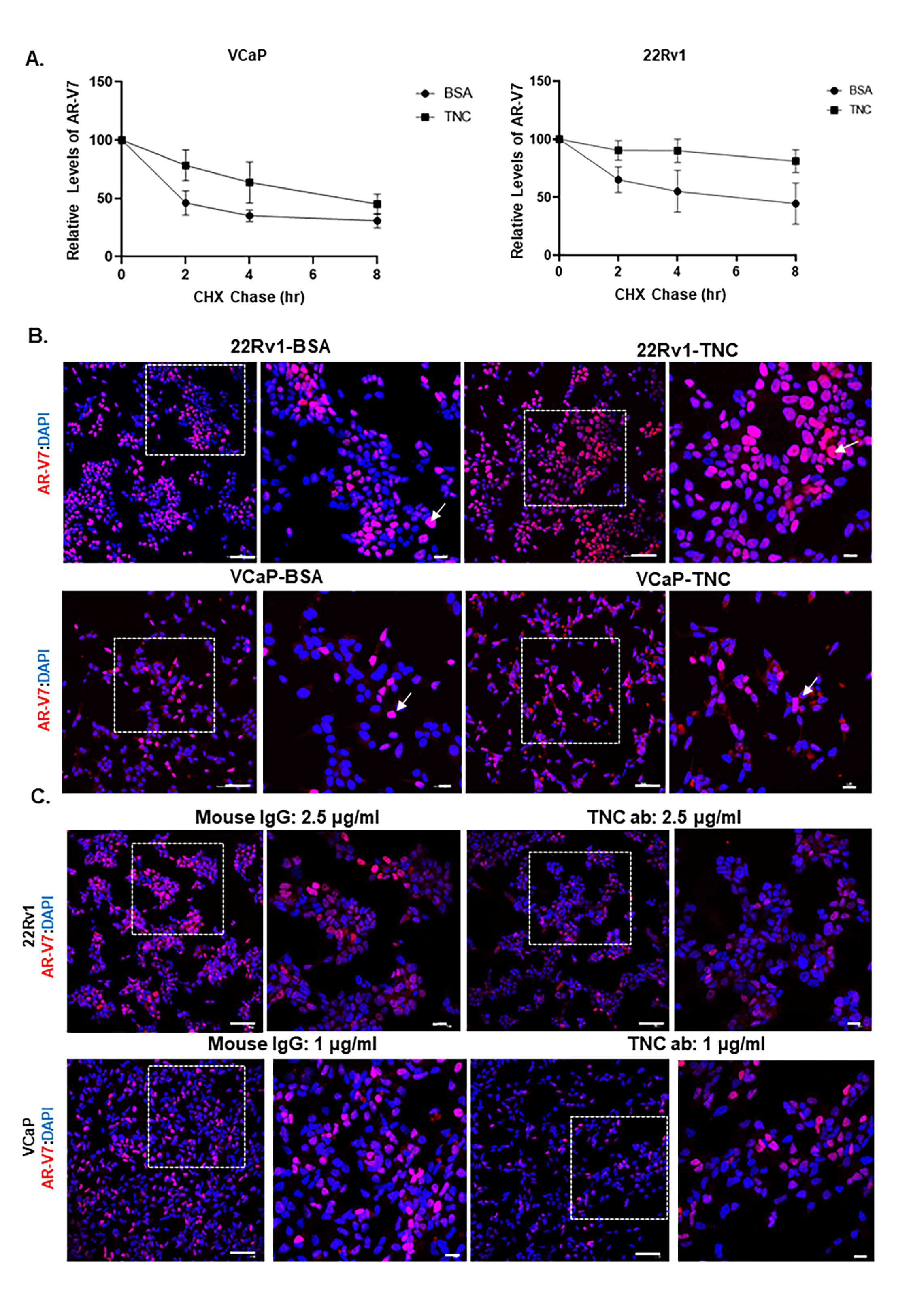

Supplement: Supplementary file 7 — Additional file 6: Fig. S4. TNC modulates AR-V7 protein stability. A VCaP and 22Rv1 plated on BSA versus TNC in 5% csFBS containing media was treated with cycloheximide. AR-V7 band intensity was normalized to β-actin and then normalized to time=0 hr (representative prior to treatment). Data represent mean ±SD for n=3 biological replicates. B. ICC of AR-V7 nuclear localization (white arrow) in both 22Rv1 and VCaP cultured on TNC compared to BSA coated IbiTreat chamber slides. The nuclei are counterstained with DAPI. All ICC images were obtained using Nikon A1 confocal microscope (Scale bar, 20x 50µm; 40x 20 µm). C. 22Rv1 and VCaP were cultured on TNC coated IbiTreat chamber slides followed by treatment with isotype control (IgG) or anti-tenascin monoclonal antibody at a concentration of 2.5 µg/ml (22Rv1) and 1 µg/ml (VCaP) respectively for 72 hours. The nuclei are counterstained with DAPI. All ICC images were obtained using Nikon A1 confocal microscope (Scale bar, 20x 50µm; 40x 20 µm). [file 12964_2022_925_MOESM7_ESM.tif]

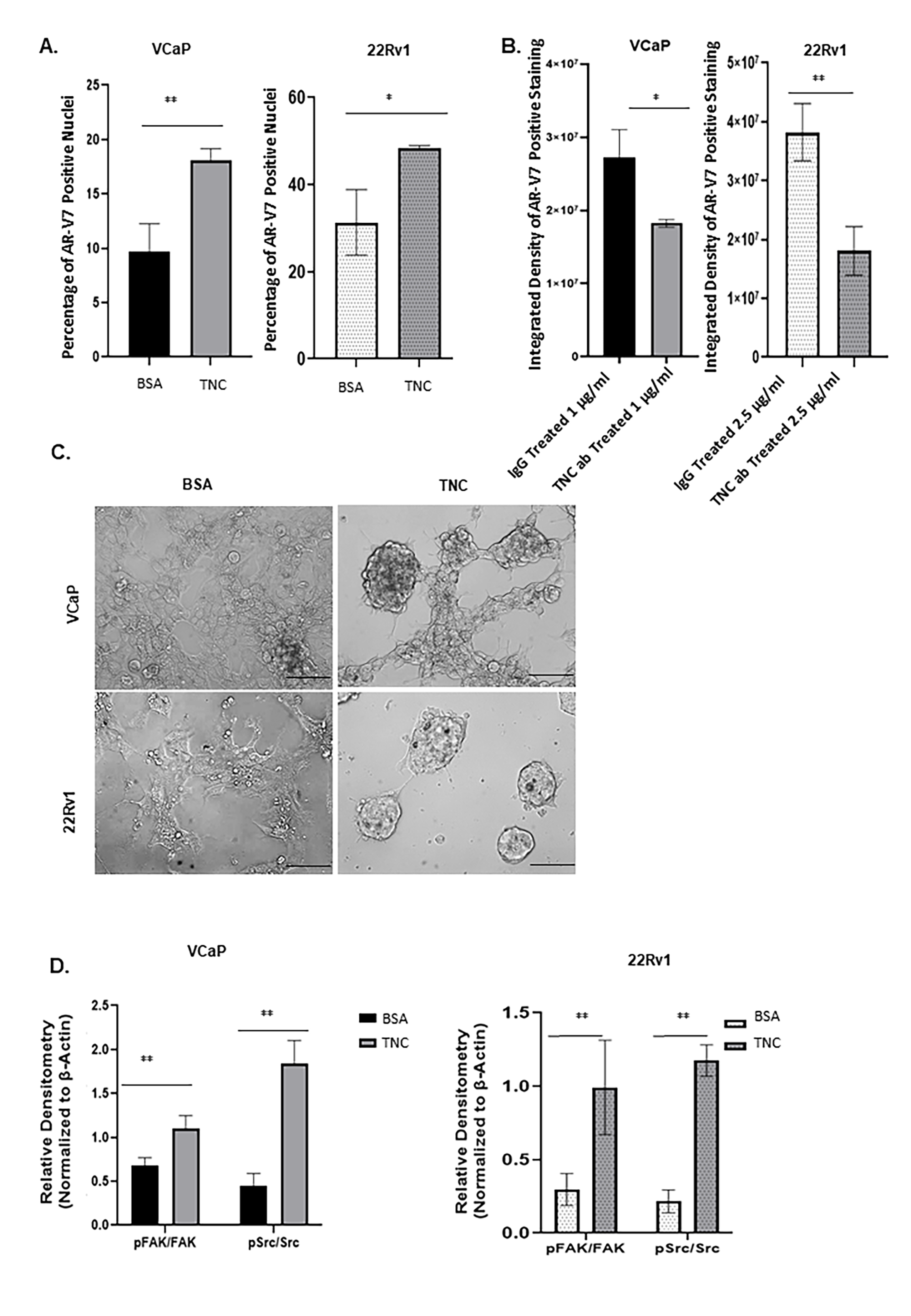

Supplement: Supplementary file 8 — Additional file 7: Fig. S5. TNC-induced increase in AR-V7 nuclear staining. A Fiji Image J quantification of AR-V7 positive nuclei in VCaP and 22Rv1 seeded on BSA versus TNC ( Scale bars, 10x: 100µm). B Fiji Image J quantification of AR-V7 nuclear intensity in VCaP and 22Rv1 seeded on BSA versus TNC (Scale bars, 10x: 100µm). C Live cell imaging of VCaP and 22Rv1 morphology seeded on BSA versus TNC using 20 x objective. D Densitometric analysis of Western blot images depicting pFAK and pSrc activation in VCaP and 22Rv1 seeded on BSA versus TNC for n=3 biological replicates. Data represent mean ± SD, n=3,*p<0.05, ** p<0.01 [file 12964_2022_925_MOESM8_ESM.tif]

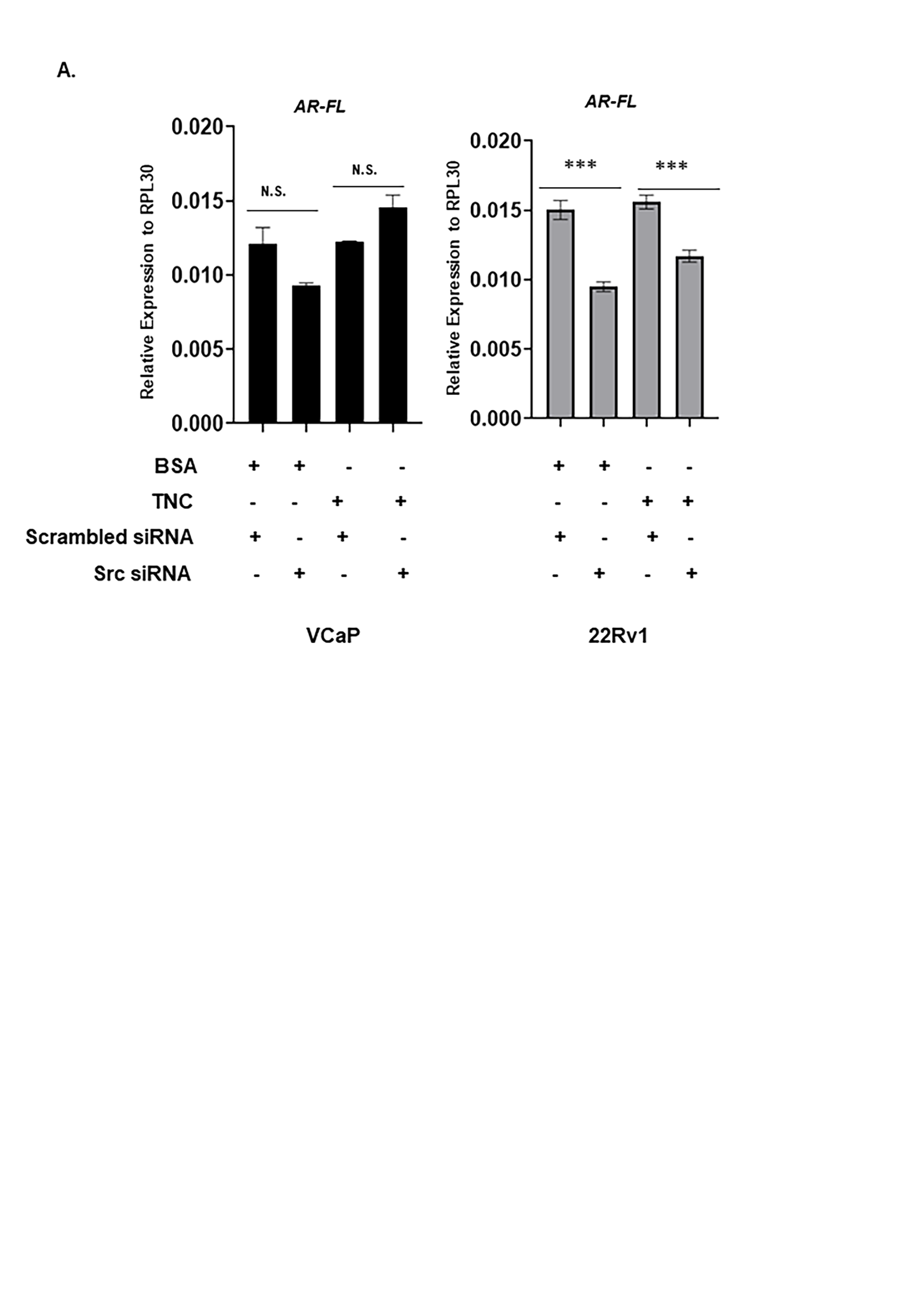

Supplement: Supplementary file 9 — Additional file 8: Fig. S6. AR-FL expression with Src knockdown. A RT-qPCR following Src knockdown in VCaP and 22Rv1 plated on BSA versus TNC. N.S represents no significances. Data represent mean ± SD, n=3, ***p<0.001. [file 12964_2022_925_MOESM9_ESM.tif]
